# Independent Regulation of Basal Neurotransmitter Release Efficacy by Variable Ca^2+^ Influx and Bouton Size at Small Central Synapses

**DOI:** 10.1371/journal.pbio.1001396

**Published:** 2012-09-25

**Authors:** Yaroslav S. Ermolyuk, Felicity G. Alder, Christian Henneberger, Dmitri A. Rusakov, Dimitri M. Kullmann, Kirill E. Volynski

**Affiliations:** UCL Institute of Neurology, University College London, United Kingdom; ICM - Institut du Cerveau et de la Moelle épinière Hôpital Pitié-Salpêtrière 47, bd de l'Hôpital, France

## Abstract

The efficacy of action potential evoked neurotransmitter release varies widely even among synapses supplied by the same axon, and the number of release-ready vesicles at each synapse is a major determinant of this heterogeneity. Here we identify a second, equally important, mechanism for release heterogeneity at small hippocampal synapses, the inter-synaptic variation of the exocytosis probability of release-ready vesicles. Using concurrent measurements of vesicular pool sizes, vesicular exocytosis rates, and presynaptic Ca^2+^ dynamics, in the same small hippocampal boutons, we show that the average fusion probability of release-ready vesicles varies among synapses supplied by the same axon with the size of the spike-evoked Ca^2+^ concentration transient. We further show that synapses with a high vesicular release probability exhibit a lower Ca^2+^ cooperativity, arguing that this is a direct consequence of increased Ca^2+^ influx at the active zone. We conclude that variability of neurotransmitter release under basal conditions at small central synapses is accounted for not only by the number of release-ready vesicles, but also by their fusion probabilities, which are set independently of bouton size by variable spike-evoked presynaptic Ca^2+^ influx.

## Abstract 2

Concurrent imaging of vesicular release and calcium dynamics in small presynaptic boutons shows that the fusion probability of readily releasable vesicles is a major determinant of the overall variability in release probability.

**Author Summary**

Synaptic transmission underlies information transfer among neurons in the brain. The probability that a synapse will release neurotransmitter in response to an action potential varies widely, even among synapses supplied by the same axon. The molecular mechanisms underlying this heterogeneity remain poorly understood. At the level of single synapses, release efficacy is determined largely by two factors: (i) the number of neurotransmitter-containing vesicles ready to be released, and (ii) by the fusion probabilities of these vesicles. By using novel imaging techniques at individual hippocampal presynaptic boutons in culture, we distinguish two independent sources of variability of release probability in small central synapses. First, we find differences in the number of releasable vesicles, and second, we find differences in the exocytosis probability of individual vesicles. To our knowledge, this is the first direct experimental demonstration that the fusion probability of release-ready vesicles is variable among synapses supplied by a single axon, and contributes roughly as much to the overall variability in release probability as does the number of release-ready vesicles.

## Introduction

The probability of neurotransmitter release (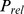) in response to an action potential (AP) determines the efficacy of information transfer at synapses in the brain. Importantly, in the central nervous system (CNS) 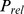 varies widely even among synapses supplied by a single axon, and this heterogeneity has been attributed in part to a target-specific adjustment of presynaptic properties [1]–[6]. However, 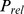 also varies considerably among synaptic boutons contacting the same type of target cell or even the same dendritic branch [2],[7]–[9], and it has been proposed that exocytosis efficacy is adjusted according to the local level of postsynaptic dendritic activity [10]. At present, however, the mechanisms that set the distinct basal release probabilities among individual small central synapses in a population remain largely unknown.

In general, 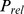 at a given synaptic bouton is determined by the number of vesicles within the readily releasable pool (RRP, vesicles docked at the active zone, AZ [11]) and by the average fusion probability (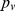) of individual RRP vesicles. According to the classical binomial model, the average number of vesicles released in response to a single AP is then 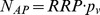 with a probability that at least one vesicle is released given by 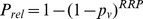. At small central synapses, both in situ and in neuronal culture, the size of the AZ, the RRP size (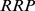), and the total recycling pool (TRP) size (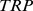) change together with the size of the presynaptic bouton [12],[13]. Thus, in general, 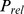 is higher at larger synapses and therefore variable presynaptic bouton size is a major determinant of 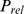. However, 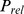 also varies substantially even among synaptic boutons of similar size (e.g., [14]–[16]). Two principal mechanisms are generally thought to underlie this heterogeneity in 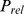. The first is variation in RRP size, due to differences in the density of docked synaptic vesicles at the AZ and/or in the dimensions of the AZ itself [12],[16]. The second mechanism that could potentially contribute to variability in 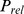 is the average fusion probability of individual RRP vesicles 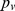, which might vary from synapse to synapse [3]. Although 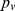 has long been known to follow changes in Ca^2+^ entry at an individual synapse, it is unclear to what extent basal 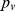 varies among boutons supplied by a given axon. Nor is it known if any systematic relationship exists between 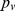 and presynaptic bouton size.

To address these fundamental questions, and to understand the relationship between the basic mechanisms that determine heterogeneity of neurotransmitter release, we have developed a suite of imaging methods that allowed us to directly relate presynaptic Ca^2+^ dynamics and vesicular exocytosis at the same small synaptic boutons. By combining fluorescence imaging of vesicular release with fluorescence imaging of presynaptic Ca^2+^ dynamics in cultured hippocampal neurons we have concurrently estimated, at the level of individual synaptic boutons, all the major functional presynaptic parameters including: the overall AP-evoked vesicular release rate 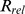, the relative sizes of the RRP and TRP, the average release probability of individual RRP vesicles 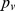, the relative bouton size, the total magnitude of presynaptic Ca^2+^ influx, and the relative endogenous Ca^2+^ buffer capacity. By comparing among boutons imaged in parallel, including those supplied by the same axon, we show that 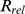, 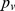, 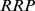, 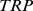, the ratio 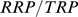, the volume-averaged AP-evoked presynaptic Ca^2+^ concentration transient 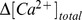, and the bouton size, all vary extensively among synapses supplied by a given axon. Whilst our results confirm that both 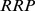 and 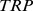 scale together with bouton size [12],[13], we find that 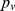 and 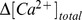 do not depend on the size of the bouton but correlate tightly with one another. Furthermore, we show that differences in 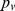 among boutons supplied by the same axon are accounted for by inter-synaptic variability in presynaptic Ca^2+^ influx but not by differences in endogenous Ca^2+^ buffering capacity. Consistent with substantial inter-bouton inhomogeneity of Ca^2+^ influx, we also show that the apparent Ca^2+^ cooperativity of AP-evoked vesicular release is lower at synapses with high 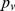. Taken together, our data argue that variable efficacies of AP-evoked neurotransmitter release among small CNS synapses supplied by a given axon are regulated by two independent mechanisms: (i) synaptic bouton size, which primarily affects the RRP size; and (ii) synapse-specific regulation of Ca^2+^ influx at the AZ, which directly determines the average fusion probability of RRP vesicles 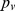.

## Results

### Co-variation of Vesicular Release Rate and AP-Evoked Presynaptic Fluorescence Ca^2+^ Transients among Individual Synapses

A major obstacle to date in relating neurotransmitter release to presynaptic Ca^2+^ dynamics is that they have not been measured together in the same small CNS synapses. To achieve this we combined two well-characterized fluorescence imaging techniques that have been extensively used in isolation: imaging of vesicular release with styryl FM dyes (e.g., [8],[17]–[19]) and measurements of presynaptic Ca^2+^ dynamics with fluorescent Ca^2+^ indicators (e.g., [20]–[24]).

**Figure 1:**
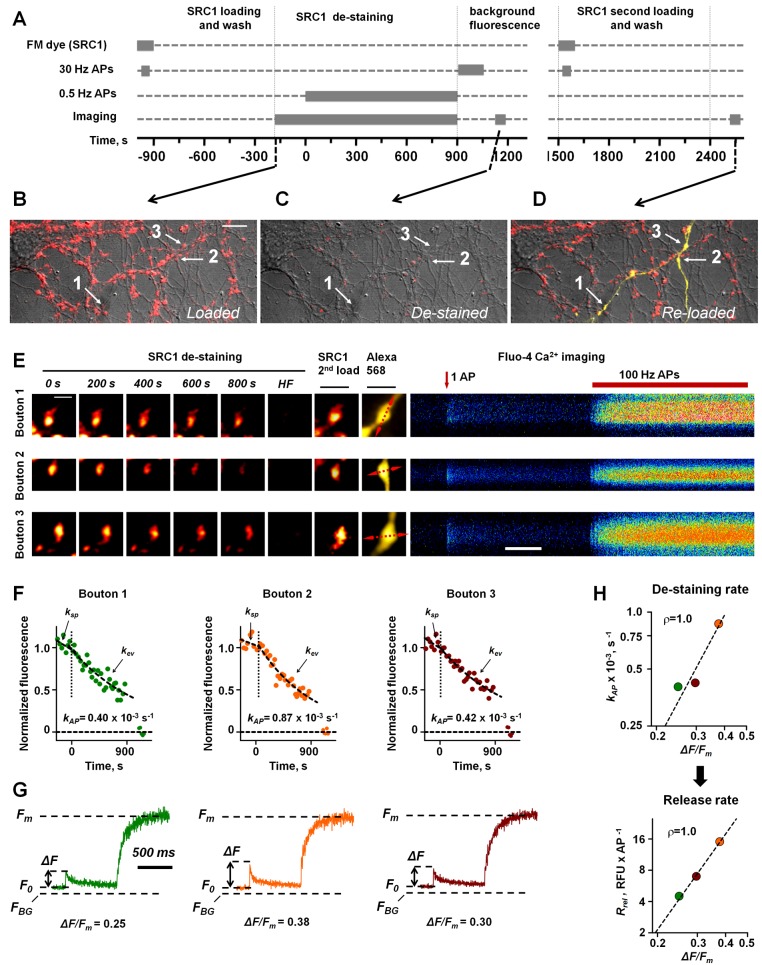
Consecutive imaging of vesicular exocytosis and presynaptic Ca^2+^ dynamics in individual synaptic boutons supplied by the same axon. (A) Experimental paradigm (sequence of loading and imaging protocols) and (B–H) a detailed illustration of a typical experiment. (B,C) Cultured hippocampal neurons (DIC and fluorescence images) showing SRC1 staining (red) before (B) and after (C) the de-staining experiment. (D) The area shown in (B) and (C), imaged after (i) re-staining with SRC1 and (ii) whole-cell patch-loading of a presynaptic cell with Fluo-4 and Alexa Fluor 568 (yellow). A single axon with two branches is clearly seen. Arrows in (B–D) depict boutons (1, 2, and 3) in which both SRC1 de-staining and Ca^2+^ dynamics have been recorded. (E) Details of SRC1 and Fluo-4 imaging in individual boutons. Left, SRC1 stained presynaptic boutons during 0.5-Hz stimulation, after the high-frequency (HF) de-staining, and after re-staining with SRC1 and patch-loading with Fluo-4 and Alexa Fluor 568. Line-scan positions used for Ca^2+^ recordings are shown with red dotted lines on the Alexa image. Right, line-scan recordings of Ca^2+^ responses in selected boutons; brightness is color-coded, red arrow, single spike onset; red segment above, a saturating 100-Hz train of APs. (F) Analysis of SRC1 de-staining in recorded boutons; fluorescence traces were normalized to the initial SRC1 fluorescence at the beginning of 0.5-Hz stimulation (marked by dotted vertical lines), dashed lines depict single-exponent fits in the absence of stimulation (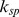) and during 0.5-Hz stimulation (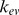). The specific AP-evoked de-staining rate in each bouton was calculated as 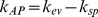. (G) Analysis of presynaptic Ca^2+^ dynamics in recorded boutons. Line-scan fluorescence time courses corresponding to stimulation paradigms shown in (E), average of five traces. Dashed lines: maximal value of Fluo-4 fluorescence *F_m_*, resting Ca^2+^ fluorescence *F_0_*, background fluorescence *F_BG_*, and peak amplitude of AP-evoked fluorescence integrated over 10 ms Δ*F.* (H) AP-evoked SRC1 de-staining rate 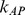 (top) and vesicular release rate 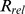 (bottom) plotted against the amplitude of AP-evoked presynaptic Ca^2+^ fluorescence *ΔF/F_m_*. Boutons are color coded as in (F) and (G). RFU, relative fluorescence units. Spearman rank correlation coefficients ρ are indicated. Dashed lines show data fits with a power function. Scale bars, 10 µm (B), 2 µm (E, top left), and 200 ms (E, bottom right).

Because Ca^2+^ indicators themselves may affect exocytosis [25],[26], we measured vesicular release prior to Ca^2+^ indicator loading. We labeled all recycling vesicles with the amphiphilic styryl dye SynaptoRedC1 (SRC1, a less lipophilic analogue of FM 4–64) using several rounds of saturating high-frequency stimulation, and recorded the SRC1 de-staining time course in individual boutons over a large area containing 300–500 putative synapses, first at rest and then during low-frequency (0.5-Hz) stimulation (Methods and Figure 1A–1C and 1E).

The SRC1 de-staining kinetics in individual boutons were well approximated by mono-exponential functions (Figure 1F). In agreement with previous reports (e.g., [14],[27]) both the spontaneous SRC1 de-staining rate determined in the absence of stimulation 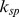, and the SRC1 de-staining rate during the 0.5-Hz AP train (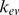) varied extensively among boutons. Furthermore, on average 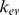
was ∼6–7-fold higher than 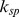 (Figure S1A and S1B). Although 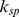 provides a measure of spontaneous exocytosis, both 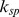 and 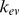 are also affected by non-specific loss of SRC1 fluorescence. To correct for these factors we calculated the specific AP-evoked SRC1 de-staining rate as 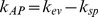. At each recorded bouton we also estimated the relative size of the TRP of vesicles as proportional to the total specific SRC1 fluorescence loss (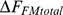) during the de-staining experiment. This was measured by calculating the difference between SRC1 fluorescence immediately after the dye washout and the residual (background) fluorescence after a series of high-frequency stimulus trains designed to release all recycling vesicles (Methods; Figure 1A). These measurements allowed us to compare AP-evoked vesicular release among individual synaptic boutons. Indeed, the specific AP-evoked vesicular release rate could be obtained from 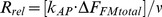 (where 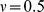 Hz is the stimulation frequency). This measure is proportional to the average number of vesicles released by a single AP, and thus closely related to 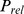 (see Discussion).

Next, we aimed to relate 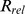 to presynaptic Ca^2+^ dynamics measured in a subset of boutons supplied by a single presynaptic neuron. To achieve this we re-loaded synaptic vesicles with SRC1 to visualize the synapses once again, and filled a nearby neuron with the high-affinity fluorescent Ca^2+^ indicator Fluo-4 together with the morphological tracer Alexa Fluor 568 via a somatic patch pipette in whole-cell mode (Methods). In approximately 5% of cases the axon of the patched cell could be traced into the area where exocytosis had previously been documented, and a subset of boutons supplied by the same axon could be identified unambiguously (Figure 1D). We then recorded presynaptic Ca^2+^ fluorescence transients in these boutons (using a 500-Hz line-scan), evoked by a single AP followed by a 100-Hz AP train (Figure 1E) to record the saturating (maximal) fluorescence of Fluo-4 [21],[22]. After subtracting the background fluorescence for each recording sweep we determined the following parameters: the resting fluorescence *F*_0_, the AP-evoked fluorescence increment Δ*F* (integrated over 10 ms), and the maximal Fluo-4 fluorescence *F_m_* (Figure 1G). To compare the magnitudes of AP-evoked presynaptic Ca^2+^ transients among individual boutons we used the ratio Δ*F/F_m_*. This allowed us to compare the amplitudes of presynaptic Ca^2+^ influx without making any assumptions about resting Ca^2+^ levels [22].

We used this protocol to measure concurrently vesicular release rates and presynaptic Ca^2+^ dynamics in two to five individual boutons supplied by the same axon (Figures 1H and S1A). Importantly, we found that in all experiments, both the AP-evoked SRC1 de-staining rate 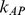, and the release rate 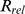, were positively correlated with the magnitude of the AP-evoked presynaptic Ca^2+^ fluorescence transient Δ*F/F_m_* (*n* = 6 independent experiments, Figures 1H and S1).

**Figure 2:**
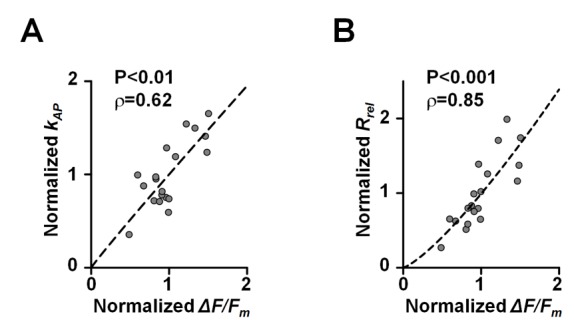
Co-variation of vesicular release rate and AP-evoked presynaptic Ca^2+^ fluorescence transients. Dependencies of AP-evoked SRC1 de-staining rate 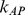 (A) and vesicular release rate 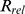 (B) on the amplitude of AP-evoked presynaptic Ca^2+^ fluorescence *ΔF/F_m_*, pooled data from six axons (*n* = 19 boutons). All parameters are normalized to the respective mean value in each experiment (axon), non-normalized data are presented in Figures 1H and S2. Correlation coefficients ρ and significance levels *p* (Spearman rank correlation test) are indicated. Dashed lines show data fits with a power function.

In addition to the magnitude of AP-evoked Ca^2+^ influx itself, the absolute value Δ*F/F_m_* is also determined by several other factors including the concentration of endogenous Ca^2+^ buffers and the equilibrated concentration of Fluo-4 in the axon [22]. These parameters may vary among different neurons ([28]; see also Figure S5A). Therefore, to combine data from boutons supplied by different axons across experiments we normalized Δ*F/F_m_*, 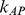, and 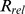 to the respective mean values in each individual cell: this normalization standardized the value range among boutons recorded in different neurons and further revealed a tight correlation between the vesicular release rate and Δ*F/F_m_* signal (Figure 2).

### Which Mechanisms Could Account for the Correlation between Δ*F*/*F*_m_ and Vesicular Exocytosis Rate?

We next asked what possible mechanisms could explain this result. The experimentally measured vesicular release rate 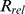 is proportional to the average number of vesicles released per AP, and therefore depends on both RRP size and 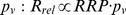. Thus the data reported above could be explained by co-variation of 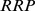 or 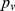, or both, with the volume-averaged AP-evoked Ca^2+^ fluorescence signal Δ*F/F_m_*.

Synaptic vesicular release is triggered by voltage-gated Ca^2+^ channels (VGCCs) that are located at the AZ in the immediate vicinity of docked RRP vesicles (for review see [29],[30]). Furthermore, accumulating functional and ultrastructural data show that VGCCs are enriched in the AZ [16],[31]–[36] where they compete to occupy specific presynaptic “slots” [37],[38] (although see [39]), which may be in a fixed stoichiometric relationship with vesicle docking sites. Therefore, it is possible that the correlation between vesicular release rates and the volume-averaged amplitude of AP-evoked presynaptic Ca^2+^ transient simply reflects inter-synaptic variability in the relative size of the AZ and/or RRP [16]. Indeed, if one of two synapses of equal volume has a larger AZ, we would expect it also to have more RRP vesicles and also more VGCCs, and therefore a higher 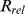 and Δ*F/F_m_*. Alternatively, the tight correlation between 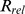 and Δ*F/F_m_* may principally reflect a different mechanism—inter-synaptic heterogeneity of AP-evoked presynaptic Ca^2+^ influx at the vesicular release sites. In this case 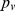 (and as a consequence 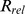) should also vary with the amplitude of the Ca^2+^ fluorescence transient.

### The Average Release Probability of Individual RRP Vesicles 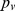 Varies Extensively among Synaptic Boutons Supplied by the Same Axon

To distinguish between these two possibilities it is necessary to compare 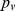 values among individual synaptic boutons supplied by the same axon. The binomial model of vesicular release (e.g., refs. [40],[41]) gives a simple expression for the SRC1 de-staining rate evoked by low-frequency AP-trains (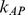), as a function of the ratio of RRP to TRP vesicular pool sizes and : 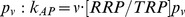 (Text S1). Therefore, to estimate 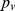 we sequentially determined the 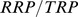 ratio and 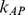 in the same boutons.

**Figure 3:**
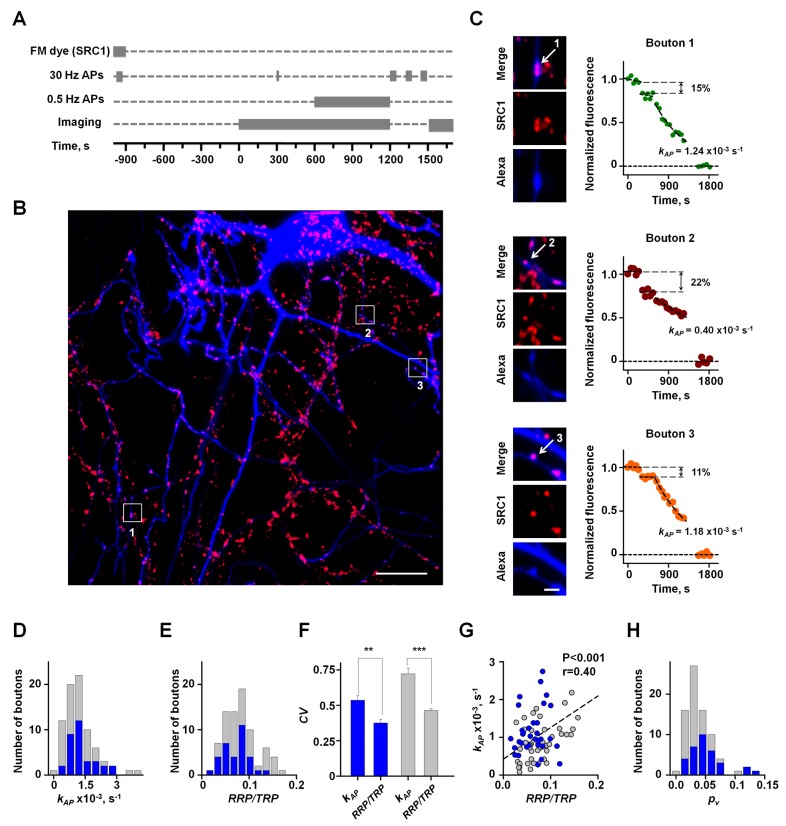
Simultaneous measurements of 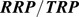 ratio and AP-evoked SRC1 de-staining rate 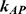 demonstrate heterogeneity of 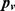 among synapses supplied by single axons. (A) Experimental paradigm. A presynaptic cell was filled with the morphological tracer Alexa Fluor 568 using whole-cell patch-clamp. Recycling synaptic vesicles in all boutons in the field of view were labeled with SRC1 using high-frequency field stimulation. After dye washout the 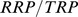 ratio in individual boutons was estimated using 30-Hz 60 AP train (see main text and Text S2 for details). This was followed by measurements of SRC1 de-staining kinetics (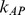) during low-frequency 0.5-Hz stimulation. (B) Fluorescence image from a typical experiment showing SRC1 labeled boutons (red) and axodentritic tree of a single neuron filled with Alexa Fluor 568 (blue). White boxes depict regions of interest containing synaptic boutons analyzed in (C). (C) High resolution images and de-staining profiles in three typical synaptic boutons (depicted by arrows) supplied by the Alexa Fluo 568 loaded neuron. (D and E) Frequency histograms of 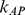 (D) and 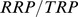 (E) from the experiment illustrated in (B and C). Blue histogram, boutons supplied by the Alexa loaded axon; grey histogram, all boutons in the field of view. (F) Comparison of 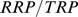 and 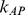 variability. Blue bars, average CVs for 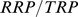 and 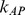 recorded in synaptic boutons located on single axons; gray bars, average CVs for the same parameters for all boutons recorded in the same experiments. Data are mean ± standard error of the mean (SEM) from 11 independent experiments, **p<0.01 and ***p<0.001, Wilcoxon signed rank test. (G) Relationship between 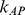 and 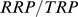. Blue, data points from boutons supplied by the Alexa loaded axon shown in (B); grey data points from all boutons in the field of view. Dotted line shows linear regression for all boutons in the field of view. Pearson's correlation coefficient *r* and significance levels *P* (Pearson product correlation test) are indicated. (H) Frequency histogram of 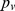 calculated for the same set of boutons as in (D, E, and G) using 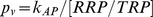. Scale bars 20 µm (B) and 2 µm (C).

The detailed experimental protocol is outlined in Figure 3A. To identify synaptic boutons supplied by a single axon we first patch-loaded a neuron in the field of view with the morphological tracer Alexa Fluo 568 (Figure 3B). After withdrawal of the patch-pipette we fully labeled the TRP of vesicles in all synapses in the field of view using saturating high-frequency field stimulation (Methods). The relative RRP size can be estimated by measuring the SRC1 fluorescence decrease evoked by brief high-frequency trains of APs that are designed to completely deplete the RRP [19],[42]–[44]. We therefore modified the classical SRC1 de-staining experiment: in addition to measuring 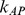 and the total specific SRC1 fluorescence loss 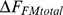 (which is proportional to the TRP size), we also measured the SRC1 fluorescence drop caused by a 30-Hz train of 60 APs 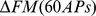 (which is proportional to the RRP size). This allowed us to estimate the 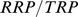 ratio as 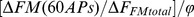 (where 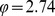 is a scaling coefficient) (see Text S2). Finally, we allowed the remaining SRC1-labeled vesicles to re-equilibrate between TRP and RRP for a further 5–7 min [19] and then measured 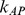 using 0.5-Hz stimulation as before (Figure 3C).

From the data thus obtained, 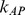, the 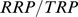 ratio, the relative RRP and TRP sizes, and the vesicular release rate 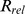, all varied extensively, even among synaptic boutons supplied by the same axon (Figures 3D–3F and S4A–S4C). In agreement with previous reports [45], RRP size correlated with the TRP size (Figure S4D), whereas the 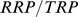 ratio was independent of the TRP size (Figure S4E). Surprisingly, even though 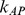 should be directly proportional to the 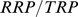 ratio, there was only a weak correlation between these parameters (e.g., Figure 3G). Indeed, some boutons with relatively low 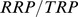 had a high AP-dependent de-staining rate (e.g., boutons 1 and 3) (Figure 3C), while other boutons with a large 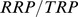 could have a relatively slow 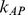 (e.g., bouton 2). Moreover, in each experiment the coefficient of variation (CV) of 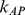 was always significantly higher than the CV of 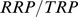 (Figure 3F). These results argue that the average fusion probability of RRP vesicles varies extensively among synapses supplied by a given axon. Indeed, by estimating 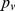 in individual synaptic boutons from 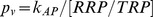 we found that the average CV of 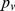 in boutons supplied by single axons was 0.65±0.05 (*n* = 11 axons, on average 16 recorded boutons/axon). Importantly the average value of 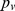 measured across all boutons (0.040±0.006) was consistent with previous estimates obtained with complementary methods [13].

Thus, we conclude that the heterogeneity of overall neurotransmitter release probability 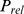 among synapses supplied by a single axon is determined not only by variability in RRP size [8],[11]–[13] but also by inter-synaptic variability in the average release probability of RRP vesicles 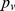.

### 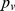 Varies among Synapses with AP-Evoked Presynaptic Ca^2+^ Fluorescence Response

**Figure 4:**
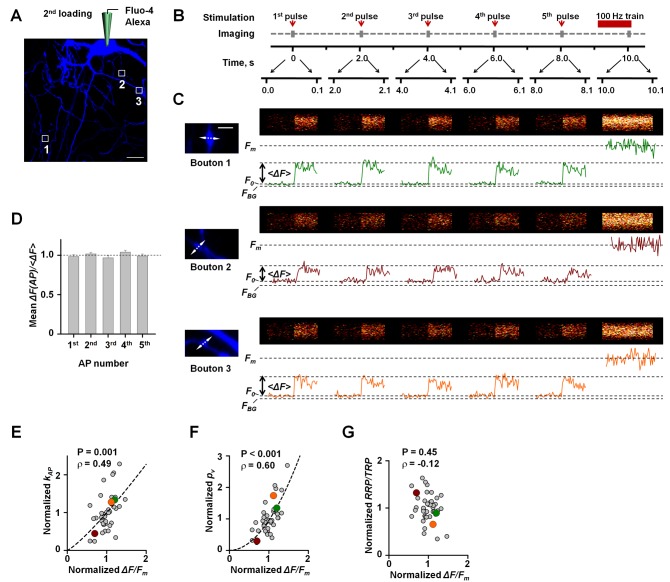
Co-variation of 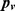 and volume averaged AP-evoked presynaptic Ca^2+^ fluorescent transient. (A) Fluorescence image (Alexa channel) of the same neuron as in Figure 3B after completion of the SRC1 de-staining experiment and subsequent re-patch-loading with Fluo-4 and Alexa Fluor 568. (B) Experimental paradigm. Ca^2+^ fluorescence responses in each bouton were recorded during a single sweep consisting of five APs delivered at 0.5 Hz followed by a saturating 100-Hz train of APs. The laser was turned on only during 100-ms intervals synchronized with single AP stimulation (brown arrows) and at the very end of the 100-Hz 100 AP train (brown horizontal bar) when Fluo-4 signal was already saturated (e.g., Figure 1G). (C) Line-scan recordings of Ca^2+^ dynamics in three typical boutons (prior to these recordings vesicular release in these boutons was measured as illustrated in Figure 3C). The morphology of each bouton (Alexa channel) and the position of line scans are shown on the left. The aspect ratio in line-scan images has been adjusted to optimize figure layout. (D) Amplitude of AP-evoked presynaptic Ca^2+^ influx does not change during 0.5-Hz stimulation. In each recorded bouton Ca^2+^ fluorescence response at every AP of the 0.5-Hz train (Δ*F*(AP)) was normalized to the average amplitude calculated for all five APs (<Δ*F*>) in the same train. The bars are the mean ± SEM values of Δ*F*(*AP*)/<Δ*F*> in all recorded boutons (*n* = 42 from ten independent experiments). Δ*F*(*AP*)/<Δ*F*> did not vary systematically with the AP number (*p* = 0.2, one-way ANOVA). (E–G) AP-evoked SRC1 de-staining rate 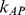 (D), the average release probability of individual RRP vesicles 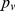 (E), and the 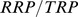 ratio (F), plotted against the amplitude of AP-evoked presynaptic Ca^2+^ fluorescence Δ*F/F_m_*. All parameters are normalized to the respective mean value in each experiment (*n* = 42 boutons from ten independent experiments). Correlation coefficients ρ and significance levels *p* (Spearman rank correlation test) are indicated. Dashed lines show data fits with a power function. Color-coded data points correspond to boutons 1, 2, and 3 from the experiment analyzed in (C) and also in Figure 3B and 3C. Scale bars 20 µm (A) and 2 µm (C).

How does the bouton-to-bouton variability in 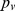 relate to differences in presynaptic Ca^2+^ dynamics? Upon completion of the SRC1 de-staining experiment we re-patched the identified neuron, loaded it with Fluo-4 and Alexa Fluor 568, and recorded presynaptic Ca^2+^ fluorescence transients in a subset of the same boutons (Figure 4). In the earlier experiments (Figure 1) we measured presynaptic Ca^2+^ dynamics in response to single APs, whilst vesicular release rates were measured during 0.5-Hz stimulation. To match the SRC1 de-staining protocol, in this set of experiments we also measured presynaptic Ca^2+^ dynamics with 0.5-Hz stimulation. For each bouton we recorded the Ca^2+^ fluorescence during a single sweep consisting of five APs delivered at 0.5 Hz followed by a saturating 100-Hz train of 100 APs. To minimise photobleaching and phototoxicity the laser was turned on only during 100-ms periods synchronized with stimulation (Figure 4B).

Consistent with the results reported in Figure 1E and 1G, the 2-s inter-pulse interval was sufficient for the Ca^2+^ fluorescence to return to its resting level (Figure 4C). Although the peak amplitude of the AP-evoked Ca^2+^ fluorescence response exhibited some variability (e.g., Figure 4C, 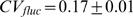, 42 boutons from ten independent experiments), there was no systematic increase or decrease of Δ*F/F_m_* during the 0.5-Hz stimulation train (Figure 4D). Our measurements of presynaptic Ca^2+^ dynamics can therefore be directly related to measurements of vesicular release rates at the same stimulation frequency. The mean Δ*F/F_m_* value calculated for all 42 boutons recorded in ten experiments was 0.34±0.02. Importantly, we observed a substantial variability in presynaptic Ca^2+^ transients among individual boutons supplied by the same neuron (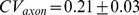). This heterogeneity could not be explained by trial-to-trial fluctuations of Δ*F/F_m_* because averaging of five trials in each bouton restricted this source of variance to only 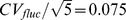, and therefore the true biological variability of presynaptic Ca^2+^ responses, expressed as CV, was 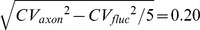.

We next related Δ*F/F_m_* to 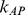, 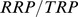, and 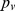 measured in the same synapses. Again we found a positive correlation between 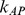 and the magnitude of AP-evoked presynaptic Ca^2+^ influx Δ*F/F_m_* (Figure 4E, pooled data from 42 boutons supplied by ten axons). Interestingly we also found that, whilst 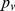 was strongly correlated with Δ*F/F_m_* (Figure 4F), the 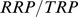 ratio did not depend on presynaptic Ca^2+^ influx (Figure 4G). Thus, these data argue that co-variation of vesicular release rate and AP-evoked Ca^2+^ fluorescence signal is a consequence of a steep dependence of the average fusion probability of release-ready vesicles on the size of the Ca^2+^ concentration transient.

### Inter-synaptic Differences in Bouton Size or Endogenous Ca^2+^ Buffering Capacity Cannot Account for Co-variation of 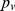 and ΔF/F_m_

The volume-averaged amplitude of the AP-evoked presynaptic Ca^2+^ fluorescence response Δ*F/F_m_* depends on three main factors: the total magnitude of presynaptic Ca^2+^ influx, the concentration and properties of endogenous presynaptic Ca^2+^ buffers, and the bouton volume. Which of these accounts for the co-variation of 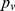 and Δ*F*/*F_m_*?

**Figure 5:**
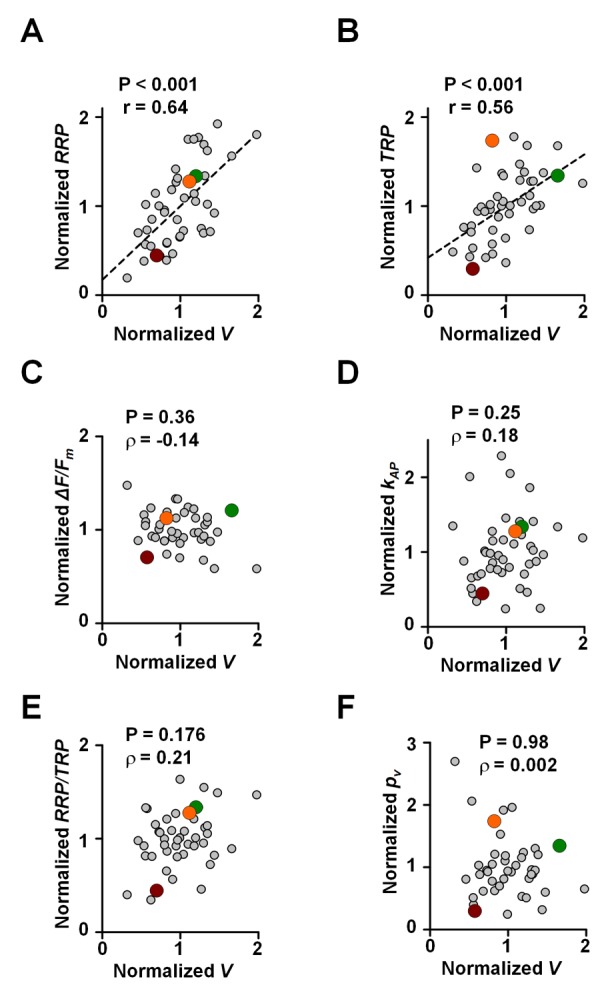
Relationships between functional presynaptic parameters and bouton size. Dependency of normalized RRP size (A), TRP size (B), AP-evoked Ca^2+^ fluorescence step Δ*F/F_m_* (C), AP-evoked SRC1 de-staining rate 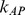 (D), the ratio 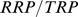 (E), and the average release of individual RRP vesicles 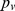 (F) on the normalized synaptic bouton volume *V*, estimated as proportional to the integral Alexa Fluor 568 fluorescence in each bouton (Figure S5C). Data are from the same set of experiments as in Figure 4E–4G (*n* = 42 boutons recorded in ten axons). All parameters are normalized to the respective mean value in each axon. Correlation coefficients ρ and significance levels *p* (Spearman rank correlation test) are indicated. Dashed lines in (A) and (B) show linear regression. Color-coded data points correspond to boutons 1, 2, and 3 from the experiment illustrated in Figures 3C and 4C. Consistent with previous reports [13] RRP size (A) and TRP size (B) increased with the synaptic bouton volume. In contrast Δ*F/F_m_* (C), 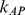 (D), 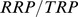 (E), and 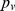 (F) did not systematically change with the synaptic bouton size.

We first estimated the relative volume of each synaptic bouton (*V*) from the Alexa Fluor 568 fluorescence (Methods; Figure S5). Consistent with electron microscopy data [11]–[13] we found that both relative RRP and TRP sizes were positively correlated with the bouton volume (Figure 5A and 5B). In contrast, we found no relation between the bouton volume and Δ*F/F_m_*, 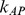, 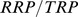, or 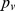 (Figure 5C–5F). Therefore, the co-variation of 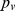 and Δ*F/F_m_* cannot be explained by the synaptic bouton volume determining both the Ca^2+^ concentration transient and the average release probability of individual RRP vesicles.

We next compared endogenous Ca^2+^ buffering among boutons supplied by the same axon. The classical steady state approximation of the single-compartment model, which is commonly used for small boutons, assumes an equilibrium between Ca^2+^ indicators and endogenous buffers, and predicts that AP-evoked Ca^2+^ transients follow a mono-exponential decay whose rate is decreased by endogenous Ca^2+^ buffers [46]–[49]. However, consistent with previous reports [20],[21],[28], we found that presynaptic Ca^2+^ fluorescence transients deviated significantly from a simple mono-exponential relaxation (Figure 1G). Most likely this was because Ca^2+^ indicators and endogenous Ca^2+^ buffers were not at equilibrium during the initial fast phase of the AP-evoked Ca^2+^ fluorescence transient [21].

**Figure 6:**
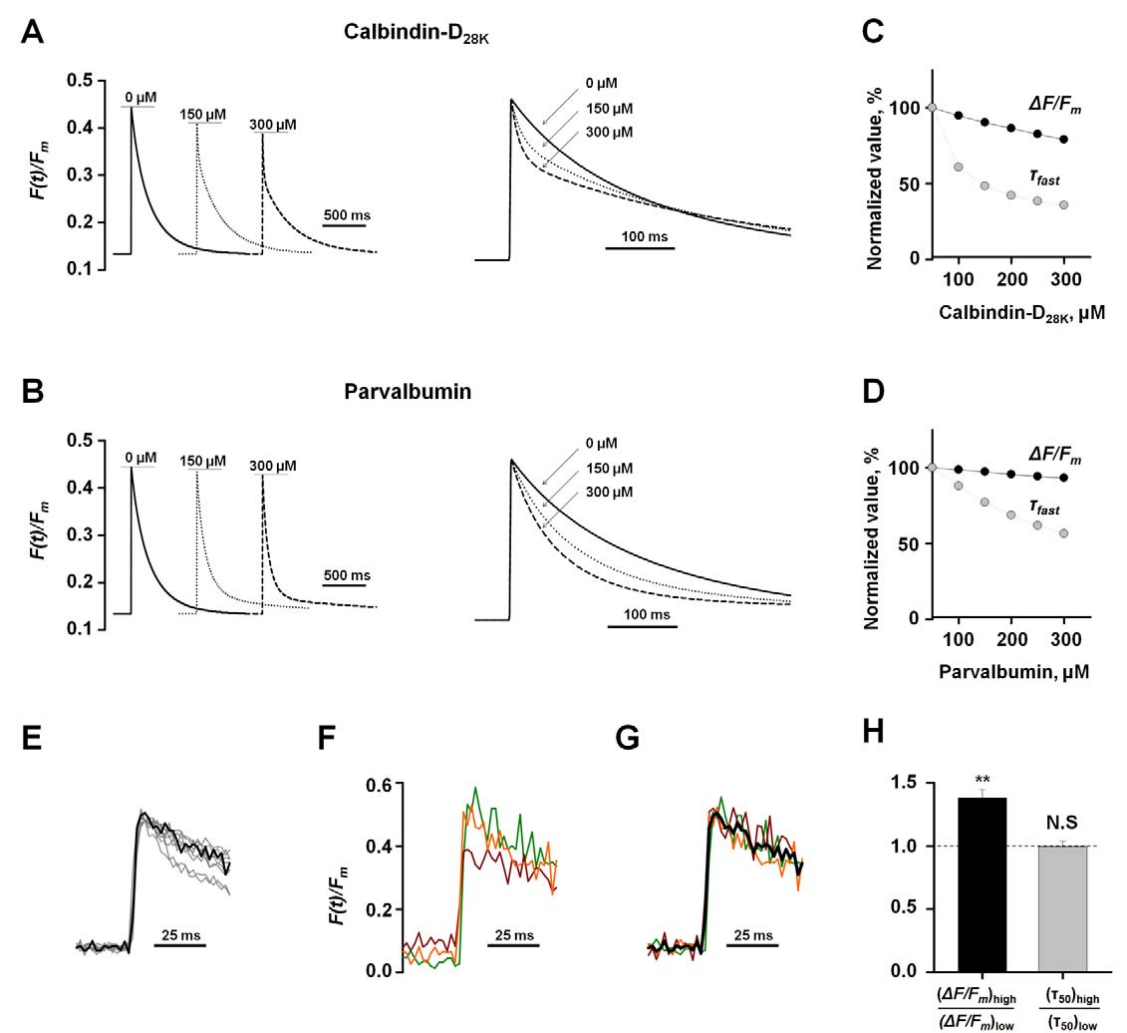
Synaptic boutons located on the same axon have similar endogenous Ca^2+^ buffering capacity. (A, B) Theoretical effects of the major endogenous neuronal Ca^2+^ buffers calbindin-D_28K_ (A) and parvalbumin (B) on presynaptic Ca^2+^ Fluo-4 fluorescence transients calculated using a non-stationary model of presynaptic Ca^2+^ dynamics (Text S3). Left, the effect of increasing concentrations of endogenous Ca^2+^ binding sites on the amplitude and the shape of fluorescence Ca^2+^ transients. Right, peak-scaled Ca^2+^ fluorescence traces illustrating an increase of the fast fluorescence decay component and a decrease of the slow fluorescence decay component with the increase of the endogenous Ca^2+^ buffer concentration. (C, D) Theoretical dependences of changes in the amplitude of AP-evoked Ca^2+^ fluorescence Δ*F/F_m_* (black) and the fast fluorescence decay time constant *τ_fast_* (grey) on intracellular concentration of calbindin-D_28K_ (C) and parvalbumin (D): *τ_fast_* is more sensitive then Δ*F/F_m_* to changes in endogenous Ca^2+^ buffering. (E) Scaled average responses to a single AP reveal heterogeneity of fast decay rates of Ca^2+^ fluorescence transients recorded in different axons (*n* = 10 experiments, the same dataset as in Figure 4E–4F). Black trace, the average response recorded in boutons 1, 2, and 3 from experiment illustrated in Figures 3C and 4C. (F, G) Detailed comparison of presynaptic Ca^2+^ dynamics in boutons 1, 2, and 3. (F) Superimposed original traces (color coded as in Figure 4C) showing variability of Δ*F/F_m_* and the resting Ca^2+^ fluorescence *F*_0_ among the three boutons. (G) Scaled responses from the same boutons showing similar fluorescence decay rates (black shows the average of three traces). (H) To test how the fast fluorescence decay rate (τ_50_, calculated by fitting the Ca^2+^ transient over 50 ms with a mono-exponential function) depends on the amplitude of presynaptic Ca^2+^ fluorescence transient, boutons recorded in each axon were divided into two groups according to the Δ*F/F_m_* value: above the median (high Ca^2+^) and below the median value (low Ca^2+^). Next, the average amplitude Δ*F/F_m_* and the average τ_50_ were calculated for each group followed by calculation of 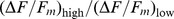 and 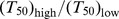. Because the ratio 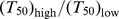
was not significantly different from 1 we conclude that fast fluorescence decay rate is the same in boutons with high and low Δ*F/F_m_*. Bars are mean ± SEM; *n* = 10 experiments, **p<0.01, non-significant (NS) *p* = 0.70, Wilcoxon singed rank test for single group median.

To compare Ca^2+^ buffering among different boutons without making any steady-state approximations we performed a numerical analysis of presynaptic Ca^2+^ dynamics using a non-stationary single compartment model [21],[24] (Text S3). We considered the effect of two major neuronal Ca^2+^ buffers, calbindin-D_28K_ and parvalbumin, on the peak amplitude Δ*F/F_m_* and on the shape of presynaptic Ca^2+^ Fluo-4 fluorescence transients (Figure 6A–6D). Within a physiological concentration range [28],[50] we found that the fast Ca^2+^ buffer calbindin-D_28K_ and the slow Ca^2+^ buffer parvalbumin have only limited effects on the peak amplitude of Δ*F/F_m_* (Figure 6A and 6C; ∼25% reduction for 300 µM calbindin-D_28K_, and ∼10% reduction for 300 µM parvalbumin). Moreover, in agreement with the experimental data (Figure 1G), Ca^2+^ fluorescence traces obtained using the non-stationary model were better described by the sum of two mono-exponential functions (*τ_fast_* and *τ_slow_*). In contrast to the steady state approximation, the non-stationary model further predicts that endogenous Ca^2+^ buffers should first accelerate and then slow down the AP-evoked Ca^2+^ fluorescence transients (Figure 6A and 6C; see also Discussion).

We then used the non-stationary model to determine which parameters of Ca^2+^ fluorescence kinetics are specifically sensitive to endogenous Ca^2+^ buffering. The Ca^2+^ buffer concentration affects the fast decay time constant *τ_fast_* to a much greater extent than the peak amplitude Δ*F/F_m_* (Figure 6B and 6D). We therefore compared the shapes of the presynaptic Ca^2+^ fluorescence transients during the first 50 ms after the AP and found that they were very similar among boutons supplied by single axons (Figures 6E–6G and S6). Moreover, the Ca^2+^ fluorescence time course did not depend on the peak amplitude Δ*F/F_m_* (Figure 6H). We therefore conclude that the observed variability in Δ*F/F_m_* among boutons supplied by the same axon cannot be explained by differences in endogenous Ca^2+^ buffering capacity. Instead, the steep correlation between pool 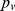 and Δ*F/F_m_* most likely reflects inter-synaptic differences in the magnitude of the volume-averaged AP-evoked presynaptic Ca^2+^ influx.

### ΔF/F_m_ Provides a Linear Readout of Presynaptic Ca^2+^ Influx

**Figure 7:**
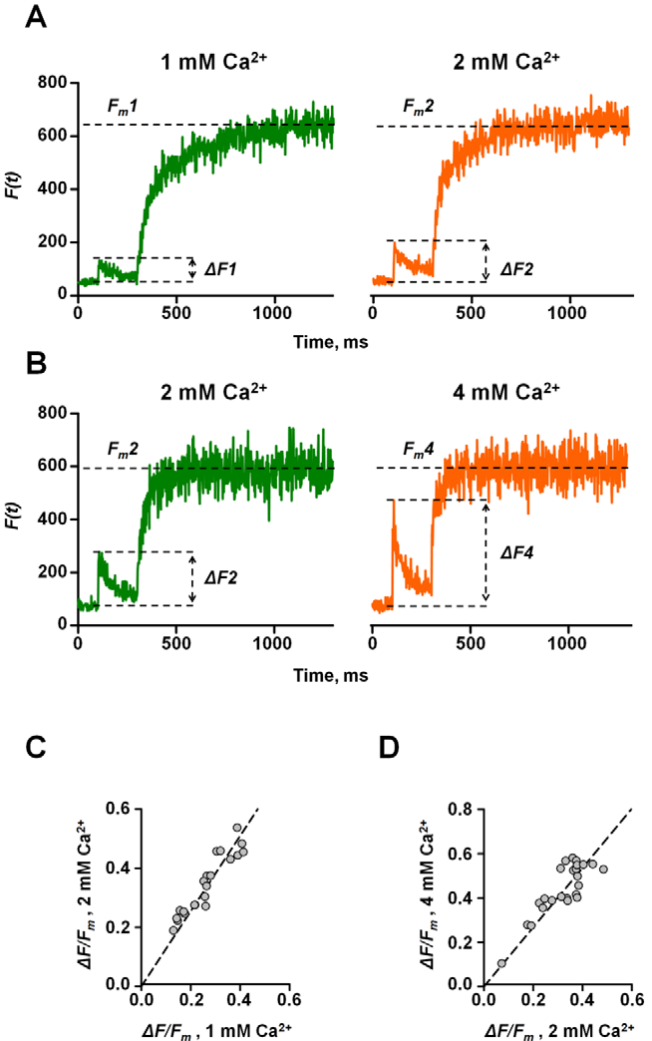
Linear relationship between ΔF/F_m_ and total magnitude of presynaptic Ca^2+^ influx. (A, B) Typical Ca^2+^ fluorescence responses to a single AP followed by a saturating train of 100 APs in individual synaptic boutons recorded at two different 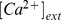: 1 mM and 2 mM (A), and 2 mM and 4 mM (B). [Mg^2+^]_ext_ was adjusted to keep the total divalent cation concentration constant (4 mM). Whilst *F_m_* measured at the end of a 100-Hz AP train was not affected by changing 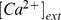, both the amplitude Δ*F* of Fluo-4 responses to a single AP and the rate of rise in fluorescence during 100-Hz stimulation were increased at higher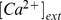. (C, D) Fractional change of peak AP-evoked fluorescence response was similar in all recorded boutons irrespective of the initial Δ*F/F_m_* value when 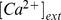 was changed from 1 mM to 2 mM (C, *n* = 24 boutons from four axons) or from 2 mM to 4 mM (D, *n* = 21 bouton from five axons). Dashed lines *y* = 1.33×(C) and *y* = 1.38×(D).

Hitherto we have assumed that Δ*F/F_m_* provides an unbiased estimate of AP-evoked Ca^2+^ influx. What is the exact relationship between the amplitude of the presynaptic Ca^2+^ fluorescence transient measured with the high affinity indicator Fluo-4 and the total magnitude of AP-evoked presynaptic Ca^2+^ influx (i.e. AP-evoked change of total intracellular Ca^2+^ concentration 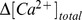)? Within the relevant range of Δ*F/F_m_* (0.1–0.6) the non-stationary single compartment model predicts a near-linear relationship between Δ*F/F_m_* and 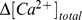 (Figure S7). To test this prediction experimentally we compared the relative changes in Δ*F/F_m_* caused by altering the extracellular Ca^2+^ concentration (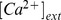) among individual synaptic boutons (Figure 7). Elevation of 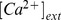 should increase presynaptic Ca^2+^ influx uniformly at all recorded boutons. On the other hand, if Fluo-4 was partially saturated following an AP the relative increase of Ca^2+^ fluorescence should be smaller in boutons with a higher initial Δ*F/F_m_* value. When 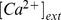 was increased from 1 mM to 2 mM, however, we observed similar proportional increases in Δ*F/F_m_* irrespective of the starting value: Δ*F/F_m_* measured at the two concentrations in different boutons fell on a straight line passing through the origin (Figure 7C, slope: 1.33±0.04). Similarly, increasing 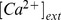 from 2 mM to 4 mM also led to similar fractional increases in Δ*F/F_m_* in all boutons (Figure 7D, slope: 1.38±0.04). We thus observed no evidence for saturation of peak fluorescence signals in our experimental conditions. We also confirmed that a 100-Hz AP train was sufficient to saturate Fluo-4 completely. Indeed, increasing 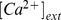 did not significantly alter the maximal fluorescence *F_m_* even in boutons with low initial Ca^2+^ influx (Figure 7A), further arguing that Δ*F/F_m_* measured with Fluo-4 provided a linear readout of 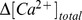.

### Ca^2+^ Cooperativity of AP-Evoked Vesicular Release Varies among Synapses

**Figure 8:**
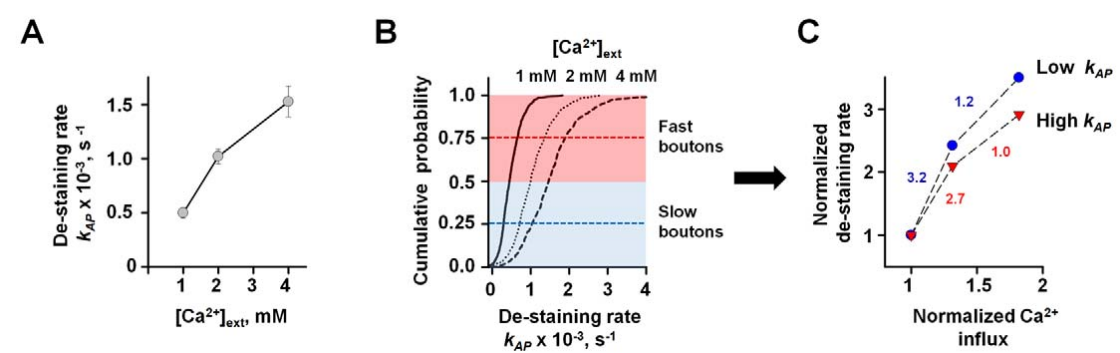
Inter-synaptic variability of Ca^2+^ cooperativity of AP-evoked vesicular release. (A) Dependency of the average AP-evoked de-staining rate 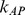 on 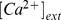. Data are mean ± SEM from six independent experiments for each 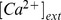. (B) Cumulative distributions of 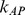 at different 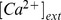
(1 mM, 620 boutons; 2 mM, 550 boutons, and 4 mM, 555 boutons). (C) Ca^2+^ cooperativity of vesicular release is higher in boutons with slow SRC1 de-staining (blue) than in boutons with fast SRC1 de-staining (red): 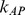 values at different 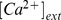 corresponding to cumulative probabilities 0.75 (Fast boutons) and 0.25 (Slow boutons) were normalized to the corresponding de-staining rates at 1 mM 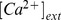 and then plotted against the relative amplitude of Ca^2+^ influx (determined as in Figure 7), which was also normalized to its value at 1 mM 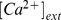. Numbers next to the dotted lines connecting the data points correspond to differential Ca^2+^ cooperativity of vesicular release calculated using the equation 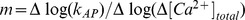 [63] (blue, slow boutons; red, fast boutons).

The predominant occurrence of VGCCs within the AZ [31],[33]–[35],[51] implies that AP-evoked 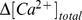 is directly related to the local Ca^2+^ concentration transients at the vesicular release sites. This further argues that the observed co-variation of 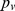 and 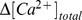 could be a direct consequence of inter-synaptic heterogeneity of local Ca^2+^ influx at the AZ. To test this prediction we measured vesicular release rates in individual synaptic boutons at 1, 2, and 4 mM 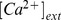. First we calculated the average 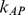 in all boutons recorded at each 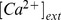. As expected, we observed a steep dependency of the average vesicular release rate on 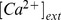 (Figure 8A). Moreover, in agreement with previous electrophysiological data (e.g., [31],[52]), we observed evidence of significant saturation of the Ca^2+^ release sensor at physiological 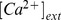: although increasing 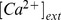 from 1 mM to 2 mM and from 2 mM to 4 mM led to similar increases in presynaptic Ca^2+^ influx 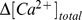 (Figure 7C and 7D), the average 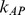 was increased ∼2.0-fold when 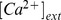 was switched from 1 mM to 2 mM, but only ∼1.5-fold when 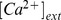 was changed from 2 mM to 4 mM (Figure 8A).

This result allowed us to make an important prediction: if inter-synaptic differences in 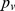 are indeed due to variability in Ca^2+^ influx at the AZ, then the Ca^2+^ release sensor should be saturated to a greater extent in synapses with higher 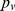. Since 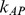 is directly proportional to 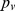 this means that Ca^2+^ cooperativity of vesicular release should be lower in synapses with fast SRC1 de-staining, and higher in synapses with slow SRC1 de-staining. To test this prediction we plotted the cumulative probability distributions for 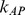 at each 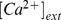 (Figure 8B) and then calculated the Ca^2+^ dependency of vesicular release rates in synapses with high and low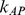. In full agreement with the above prediction we found a much steeper dependency of vesicular release rates on presynaptic Ca^2+^ influx in synapses with low 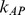 (Figure 8C).

We also considered an alternative explanation for this result. Although vesicles docked at the same AZ have been reported to exocytose independently of one another [40],[41],[53], some studies have argued that vesicular release in small central synapses is restricted to only one vesicle per AZ per AP [7],[54]. According to the latter model synapses with high 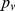, and as a consequence high 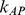, should indeed be less sensitive to changes in 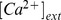 [8]. But then 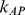 should also be considerably lower in larger synaptic boutons, which contain more readily releasable vesicles. However 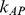 did not vary systematically either with the RRP and TRP size (Figures S1F, S4F and S4G) or with the synaptic bouton size (Figure 5D). Our results therefore argue that the inverse relationship between the AP-evoked SRC1 de-staining rate and the Ca^2+^ cooperativity of release is a direct consequence of inter-synaptic variability of local Ca^2+^ influx at the AZ.

## Discussion

The goal of this study was to understand the major factors underlying heterogeneity of AP-evoked neurotransmitter release at small central synapses. It is commonly accepted that the efficacy of synaptic vesicular release is directly determined by the number of release-ready vesicles which, in turn, is related to the AZ size and the presynaptic bouton volume [8],[13],[15],[16],[55]. By measuring exocytosis and Ca^2+^ signaling concurrently, and by dissecting 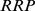 and 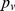 at individual boutons, we have identified a second major determinant of different release probabilities among synapses: the average fusion probability of individual RRP vesicles 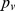. Our data argue that 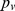 varies as a direct consequence of inter-synaptic differences of AP–evoked presynaptic Ca^2+^ influx independently of 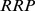 and synaptic bouton size. Both sources of variability, 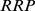 and 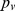 contribute approximately equally to the overall variability of 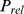 among synapses supplied by the same axon.

Current understanding of the quantitative relationship between presynaptic Ca^2+^ entry and neurotransmitter release relies mostly on studies of individual large or giant synapses, such as those made by hippocampal mossy fibers or the Calyx of Held [36],[56],[57], which can be patch-clamped to control the presynaptic membrane potential and to manipulate or measure Ca^2+^ concentrations. However, the overwhelming majority of synapses in the CNS are too small (∼1 µm scale) to permit such approaches, and therefore until now, the Ca^2+^ dependency of neurotransmitter release in small synaptic boutons could only be assessed at the population level, thus obscuring the determinants of heterogeneity (for review see [30],[58],[59]). We have overcome this barrier by combining imaging of vesicular exocytosis with measurements of presynaptic Ca^2+^ dynamics at the same small boutons.

To compare the efficacy of AP-evoked vesicular release among synapses supplied by a given axon we modified the classical FM-dye imaging approach and simultaneously measured, at the level of single boutons, all the major determinants of AP-evoked vesicular release including RRP size, the average fusion probability of individual RRP vesicles 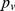, and the overall AP-evoked vesicular release rate 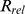. It should be noted that whilst 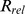 is directly proportional to the average number of vesicles released in response to a single AP (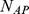), it is not exactly the same as a common interpretation of 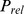, which is the probability that at least one vesicle is released during a single AP. Because 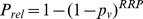, it follows that 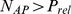 unless 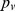 is small [8],[41]. However, because in our dataset average 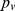 = 0.04 (Figure 3), and the RRP in cultured hippocampal neurons contains on average five to seven vesicles [12],[13], we estimate that the average values for 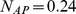 and 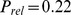 were indeed similar.

The methodological advances reported here include not only the first simultaneous measurements of RRP size and 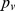 at small synapses supplied by a single axon, but also a validation of the use of the high-affinity Ca^2+^ indicator Fluo-4 for an unbiased read-out of presynaptic Ca^2+^ dynamics. It has been previously shown that Ca^2+^ fluorescence decay time constants (which are thought to be proportional to the endogenous Ca^2+^ buffering capacity) can vary among putative synaptic boutons in the neocortex [20] or in the cerebellum [49]. However, we did not find any significant differences in the time-course of AP-evoked Ca^2+^ fluorescence transients measured with the high affinity Ca^2+^ indicator Fluo-4 in boutons supplied by single axons of cultured hippocampal neurons. This argues that inter-bouton variability of Δ*F/F_m_* signals observed in our experiments cannot be simply explained by differences in endogenous Ca^2+^ buffers. Indeed, a non-stationary single-compartment model of presynaptic Ca^2+^ dynamics predicts that, whilst endogenous Ca^2+^ buffers should substantially affect the kinetics of AP-evoked Ca^2+^ fluorescence transients, they should exert only limited effects on peak Δ*F/F_m_* values (Figure 6). This at a first sight unexpected result can be explained if one bears in mind that the Ca^2+^ association rate of the fast BAPTA-based Ca^2+^ fluorescent dye Fluo-4 is 10–30-fold higher than that of the major endogenous Ca^2+^ buffers such as parvalbumin and calbindin-D_28K_ (Table S1). Therefore Ca^2+^ ions entering presynaptic bouton during an AP should first bind to the fast Ca^2+^ indicator Fluo-4 and only then to the slower acting buffers such as calbindin-D_28K_ or parvalbumin. This also explains deviation of AP-evoked Ca^2+^ fluorescence profiles from the monoexponential kinetics predicted by a classical steady-state model [46]–[49], arguing against the common assumption that endogenous Ca^2+^ buffers uniformly slow down the decay of AP-evoked Ca^2+^ fluorescence transients. In fact, we find that endogenous buffers first accelerate and then slow down the AP-evoked Ca^2+^ fluorescence kinetics (Figure 6A and 6C).

Another interesting prediction from the non-stationary analysis was that Δ*F/F_m_* should give a linear readout of the total magnitude of the AP-evoked presynaptic Ca^2+^ influx 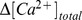 (providing that 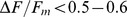) (Figure S7). Again, at a first sight this was unexpected, because it is commonly thought that high affinity Ca^2+^ indicators (e.g., Fluo-4) do not linearly report AP-evoked changes in presynaptic Ca^2+^ concentration. This is indeed the case when one considers changes in the *free* cytosolic Ca^2+^ concentration 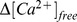 [20],[22],[49]. However, the total AP-evoked change of in presynaptic Ca^2+^ concentration also includes changes in the concentration of Ca^2+^ bound to Fluo-4 and Ca^2+^ bound to endogenous Ca^2+^ buffers: 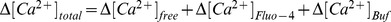. The model then predicts that, when the total intracellular concentration of Fluo-4 is sufficiently high, a large part of the Ca^2+^ entering the nerve terminal during an AP should bind to the indicator (Figure S7, 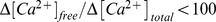), and therefore that 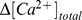 should be proportional to Δ*F/F_m_*. In full agreement with this prediction we found that Δ*F/F_m_* was indeed proportional to 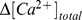 when we manipulated extracellular [Ca^2+^] (Figure 7).

Importantly, the above results argue that the major experimental paradigm used here (where SRC1 measurements of vesicular release were performed prior to Fluo-4 loading) provides an unbiased comparison of Ca^2+^-exocytosis coupling among individual boutons supplied by a given axon. Indeed whilst the absolute Δ*F/F_m_* value in each experiment does depend on the total intraterminal concentration of Fluo-4, the intracellular loading of Fluo-4 does not affect the AP-evoked Ca^2+^ influx itself. Furthermore, because the Fluo-4 concentration was the same in all boutons supplied by the same axon (Figure S5), and because Δ*F/F_m_* was proportional to 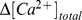, inter-synaptic differences in Δ*F/F_m_* should be proportional to differences in 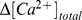 that would have been observed even in the absence of Fluo-4.

The strong correlation between Δ*F/F_m_* and vesicular release rates further argues that conventional fluorescence measurements of volume-averaged presynaptic Ca^2+^ transients provide a faithful gauge of the vesicular release probability, at least within a homologous population of synapses. Indeed, the predominant location of presynaptic VGCCs at the AZ [31]–[35] suggests that Δ*F/F_m_* should be determined mainly by Ca^2+^ influx at the AZ. On the other hand the total number of Ca^2+^ ions entering a presynaptic terminal during an AP is proportional to Δ*F/F_m_* and to the bouton volume: 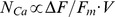. Then, by bearing in mind that in general the size of the RRP is proportional to the bouton volume (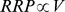) (Figure 5A) [12], we infer that the average magnitude of AP-evoked Ca^2+^ influx calculated per single docked vesicle (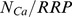) should be directly proportional to the AP-evoked Ca^2+^ fluorescence signal: 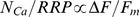. In full agreement with this prediction we found that the Ca^2+^ cooperativity of vesicular release was significantly lower in synaptic boutons with high 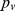 (Figure 8), which points to relative saturation of the Ca^2+^ vesicular release sensor due to higher Ca^2+^ influx at the AZ.

Our main finding is that both 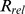 and 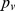 co-vary with the AP-evoked Ca^2+^ fluorescence response Δ*F/F_m_*, arguing that inter-synaptic variability of presynaptic Ca^2+^ dynamics is a major determinant of bouton to bouton differences in synaptic efficacy. Vesicular release probability may also be regulated by other factors such as small differences in endogenous Ca^2+^ buffering, which could not be detected with high concentrations of Fluo-4, or differences in the coupling distance between VGCCs and Ca^2+^ sensors for exocytosis [39]. However, if these factors play an important role in setting the basal 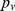 in a particular neuron they are unlikely to do so differentially at the level of individual boutons supplied by a given axon, because such variability would be expected to disrupt the tight correlation observed between 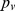 and Δ*F/F_m_* in the present study.

What are the possible mechanisms that could account for the heterogeneity of Ca^2+^ influx? AP-dependent release at small hippocampal synapses is triggered by mixed populations of P/Q- and N-type VGCCs that have been proposed to saturate specific presynaptic “slots” [37],[38] (although see [39]). These hypothetical VGCC slots put a limit on the number of VGCCs that can trigger release of docked vesicles in the RRP. Indeed, opening of three or fewer P/Q-type VGCCs appears to be sufficient to trigger release at GABAergic hippocampal synapses [60]. Moreover, the recent finding that RIM proteins specifically tether P/Q- and N-type channels to the AZ provides a possible mechanism for the molecular organization of such VGCC-specific slots [34],[35]. This raises the possibility that the average number of presynaptic slots (and as a consequence the number of active VGCCs) surrounding vesicles in the RRP might vary among presynaptic boutons located on the same axon. According to this model, the number of VGCCs per RRP vesicle would provide a straightforward mechanism for direct regulation of 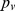. An alternative hypothesis is that heterogeneity in Ca^2+^ influx reflects systematic differences in the probability of VGCC opening during an AP potential or in the Ca^2+^ flux via individual channels. This in turn could be caused by several factors including inter-synaptic differences in the AP shape, differences in the type-specific composition of presynaptic VGCC mix [61], differential regulation of VGCC by G-proteins [62] or by auxiliary subunits [39]. However, which of these factors account for the variability of presynaptic Ca^2+^ influx cannot be determined at present.

Parallel regulation of AP-evoked vesicular release by RRP size and by Ca^2+^-dependent scaling of 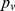 has important physiological implications, because it potentially allows the basal strength of synaptic transmission to be dissociated from short-term synaptic plasticity. For instance, during a train of presynaptic APs, boutons with a high basal 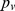 should exhibit more use-dependent depression because of depletion of the RRP, and/or attenuated facilitation because of saturation of the Ca^2+^ sensor for exocytosis. Conversely, if 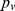 were similar at boutons of different size, and therefore different RRP size, synapses with high and low basal release probability should exhibit similar short term plasticity (assuming, that is, that vesicles in the RRP fuse independently of one another). Furthermore, Ca^2+^-dependent regulation of 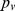 should also allow synapse-specific modulation of neurotransmitter release that depends on changes in AP-evoked presynaptic Ca^2+^ influx: stronger saturation of the Ca^2+^ sensor for exocytosis in boutons with high 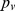 should make vesicular release in these synapses less sensitive to changes in presynaptic Ca^2+^ influx, due for example to modulation of VGCCs by G-proteins. Thus our data argue that inter-synaptic variability of Ca^2+^ influx provides a direct mechanism for the regulation and use-dependent redistribution of synaptic strengths across populations of small central synapses.

## Methods

### Cell Cultures and Imaging Solutions

Hippocampal neurons were isolated from P0–P2 rat pups [14] and cultured in Neurobasal based medium on an astrocyte feeder layer. All experiments were conducted at ambient temperature (23°C to 26°C) 13–19 d after plating. The imaging solution contained (in mM) 125 NaCl, 2.5 KCl, 2 MgCl_2_, 2 CaCl_2_, 30 glucose, and 25 HEPES (pH 7.4). To avoid recurrent activity synaptic transmission was blocked by supplementing the imaging solution with (in µM) 10 NBQX (Ascent Scientific), 50 DL-AP5 (Ascent Scientific), and 100 Picrotoxin (Tocris Bioscience).

### Fluorescence Set-Up

Fluorescence imaging experiments were performed on an inverted LSM 510 confocal microscope (Zeiss) using a 63× (1.4 NA) oil immersion objective. Fluo-4 and SRC1 were simultaneously excited using the 488-nm line of an argon laser and emissions were recorded using band pass 505–550-nm and long pass 585-nm filters, respectively. Alexa Fluor 568 was excited by a 543-nm He-Ne laser and its emission was recorded using a band pass 560–615-nm filter. Cross-talk between the fluorescence channels was less than 5%.

### Imaging of Vesicular Release

To image AP-evoked vesicular exocytosis all recycling vesicles were labeled with the low affinity red fluorescence styryl dye SRC1 (SynaptoRed C1, Biotium) (at a bath concentration of 200 µM) using several round of exhaustive high-frequency stimulation (four trains of 120 APs at 30 Hz delivered at 20-s intervals) followed by dye washout for 15 min with the imaging solution. APs were evoked by field stimulation via platinum bath electrodes separated by 1 cm (12.5–15 V, 1-ms pulses). The SRC1 fluorescence decay during high-and low-frequency stimulations was recorded as detailed in experimental paradigms (Figures 1A and 3A) in a ∼150 µm×150 µm region of interest (ROI; 1,024×1,024 pixels) containing several hundred boutons. Background fluorescence in the SRC1 channel was determined by applying three rounds of high-frequency stimulation used for loading. The SRC1 photobleaching rate determined in control experiments was less than 0.2% per frame.

Images were analyzed using ImageJ (US National Institutes of Health). Following the X-Y alignment of recorded SRC1 consecutive images, active boutons were identified by subtracting a five-frame background average (acquired after the high-frequency de-staining stimulation protocol) from a five-frame average immediately after completion of SRC1 washout. To measure SRC1 de-staining kinetics in individual synaptic boutons fluorescence intensity was obtained from ROIs centered at the fluorescence maxima of individual boutons that completely covered the bouton area (characteristic linear dimensions ∼1.0–2.0 µm). Boutons with overlapping ROIs were excluded from the analysis. The spontaneous (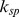) and AP-evoked (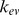) de-staining rates were calculated by fitting mono-exponential functions to the fluorescence time course in each selected ROI, after subtracting the background value. The specific AP-evoked SRC1 de-staining rate was calculated as 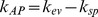. Boutons with high spontaneous de-staining rate (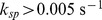) and boutons with low signal to noise ratio (goodness of the fit 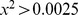) were excluded from the analysis. The size of the TRP of vesicles was estimated as proportional to the total specific fluorescence loss in each ROI: 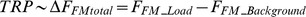 (where 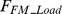 is a five-frame average of SRC1 fluorescence immediately after completion of SRC1 washout and 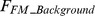 is a five-frame background average acquired after the high-frequency de-staining stimulation protocol). The size of the RRP of vesicles was estimated as proportional to the total fluorescence loss triggered by a 30-Hz train of 60 APs: 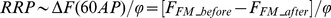 (where 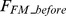 and 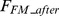 are SRC1 fluorescence measured immediately before and after the train and 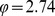 is a scaling factor determined as detailed in Text S2).

### Imaging of Presynaptic Ca^2+^ Dynamics

For presynaptic Ca^2+^ imaging the selected neuron was loaded, via a whole-cell pipette, with 200 µM Fluo-4 and 200 µM Alexa Fluor 568 in a solution containing (mM): 135 KMS, 10 HEPES, 10 Na-phosphocreatine, 4 MgCl_2_, 4 Na_2_-ATP, 0.4 Na GTP. After breaking in, the fluorescence in the soma and the apical dendrites equilibrated within 5 min and the patch pipette was slowly withdrawn to minimize cytosol dialysis. Experiments were terminated if the resting membrane potential was above −55 mV, or if the gigaseal was lost during pipette withdrawal. The dyes were allowed to equilibrate throughout the neuron for 40–50 min after retracting the patch pipette (Figure S5A and S5B), before Ca^2+^ fluorescence recordings were started. Fluorescence transients in identified boutons were recorded in fast line-scan mode (∼500 Hz). Neurons were first stimulated using field electrodes with either a single pulse (five trials for each bouton) (Figures 1 and 7) or with five pulses at 0.5 Hz (Figure 4) to determine the magnitude of AP-evoked presynaptic Ca^2+^ transients, and then by a burst of high-frequency stimulation (100 APs at 100 Hz) to determine the maximal fluorescence of saturated Fluo-4 [21],[22],[25]. After averaging of five individual trials and subtracting the background fluorescence in the Fluo-4 channel (which was determined outside of the path-loaded neuron) the amplitude of AP-evoked Ca^2+^ fluorescence responses in each bouton Δ*F/F_m_* was calculated as outlined in Figure 1G. The relative bouton sizes (cytosol volume *V*) were estimated by calculating the specific Alexa Fluor 568 fluorescence in the corresponding ROIs as outlined in Figure S5C.

## Supporting Information

Figure S1
**Properties of SRC1 de-staining in individual synaptic boutons during low-frequency stimulation.** (A–E) Distribution of SRC1 de-staining parameters recorded in individual boutons (309 boutons from five independent experiments). (A) De-staining rate in the absence of stimulation, 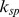; (B) de-staining rate during 0.5-Hz stimulation, 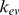; (C) Specific AP-evoked de-staining rate 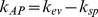; (D) Relative size of recycling pool of vesicles, calculated as 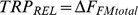 (RFU, relative fluorescence units); (E) Vesicular release rate, 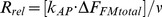 (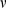 = 0.5 Hz, stimulation frequency). (F) The AP-evoked de-staining rate 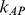 does not depend on the TRP size. Correlation coefficient ρ and significance level *p* (Spearman rank correlation test) are indicated. (G–I) Specific SRC1 de-staining rates calculated per AP are the same during 0.25-Hz and 0.5-Hz stimulation. (G) Average de-staining profiles from two typical experiments at 0.5-Hz and 0.25-Hz stimulation. Distributions of AP-evoked SRC1 de-staining rates at 0.5 Hz (black, 410 boutons from four experiments) and at 0.25 Hz (gray, 544 boutons from four experiments) calculated per second (H) or per AP (I). The absolute AP-evoked SRC1 de-staining rate was lower during 0.25-Hz stimulation (H), whilst the specific SRC1 de-staining rate calculated per AP had the same distribution at 0.25 Hz and 0.5 Hz (I), *p* = 0.28, Kolmogorov-Smirnov test.(TIF)

Figure S2
**Co-variation of vesicular release rate and AP-evoked presynaptic Ca^2+^ fluorescence transient: raw data from single axons.** Dependencies of AP-evoked SRC1 de-staining rate 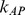 (top row) and vesicular release rate 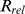 (bottom row) on the amplitude of AP-evoked presynaptic Ca^2+^ fluorescence Δ*F/F_m_* in five axons measured in five independent experiments. SRC1 measurements were performed prior to Fluo-4 loading (experimental protocol as in Figure 1, pooled normalized data are shown in Figure 2). Spearman rank correlation coefficients ρ are indicated. Dashed lines show data fits with a power function 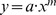.(TIF)

Figure S3
**Verification of the protocol for estimating of** 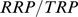 **ratio with short high-frequency stimulation bursts (related to Text S2).** (A) Experimental paradigm. After SRC1 loading and washout relative SRC1 fluorescence losses in individual boutons were determined after two trains of 30-Hz stimulation separated by a 7.5-s interval. (B) Example traces recorded in individual boutons using different stimulations protocols. (C) Dependency of the ratio 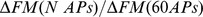 between average SRC1 fluorescence losses after the test train (consisting of *n* APs) and control train (consisting of 60 APs) on the number of APs in the test train. Dashed line represents a least square data fit using Equation 2.5 from Text S2. Data are mean ± SEM from 150–300 individual boutons from four independent experiments for each condition.(TIF)

Figure S4
**Comparison of functional vesicular pool sizes in synapses supplied by single axons.** (A–C) Left panels, frequency histograms of: (A) relative TRP size calculated as total specific loss of SRC1 fluorescence during de-staining experiment: 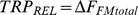, (B) relative RRP size calculated as the specific fluorescence loss stimulated by a 2-s 30-Hz stimulation divided by the scaling coefficient 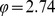 (see Text S2) 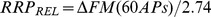, and (C) relative vesicular release rate 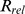 calculated as 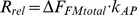 from the experiment illustrated in (Figure 3). Blue histogram, boutons supplied by the Alexa loaded axon; gray histogram, all boutons in the field of view. (A–C) Right panels, summary data for variability of: (A) TRP size, (B) RRP size, and (C) 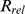. Blue bars, average CVs for synaptic boutons located on single axons; grey bars, average CVs for all boutons recorded in the same experiments. Data are mean ± SEM from 11 independent experiments. (D, E). RRP size scales linearly with the TRP size: relationships between 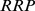 and 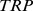 (D) and between 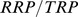 and 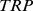 (E). (F, G) AP-evoked SRC1 de-staining rate 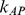 does not depend on the RRP size (F) or on the TRP size (G). Data in (D–G) are from the same experiment as illustrated in Figure 3. Blue, data points from boutons supplied by the Alexa loaded axon; grey data points from all boutons in the field of view. Dotted lines in (D–G) show linear regression for all data points. Correlation coefficients ρ and significance levels *p* (Spearman rank correlation test) are indicated.(TIF)

Figure S5 **Patch-loading of synaptic boutons with the Ca^2+^ indicator Fluo-4 and the fluorescent morphological tracer Alexa Fluor 568 and measurements of synaptic bouton size.** (A) Fluorescence image of a typical cultured hippocampal neuron 45 min after the beginning of patch-loading with Alexa Fluor 568 and Fluo-4. Note that the patch pipette was withdrawn within 5 min to minimize cytosol dialysis (Methods). The Alexa channel is shown in red, the Fluo-4 channel is not shown, and a reconstructed axon is shown in yellow. Inserts illustrate fluorescence images recorded in axonal boutons located at different distances from the soma (white boxes, ROIs 1, 2, and 3) at 5 min, 15 min, and 45 min after establishing of the whole-cell recording. Scale bars: main figure 20 µm, inserts 2 µm. (B) Time course of Alexa Fluor 568 fluorescence in the selected ROIs from (A). The gray box highlights the interval of the patch-loading (5 min). Since Fluo-4 and Alexa Fluor 568 have indistinguishable intracellular diffusion rates [21] we used Alexa Fluor 568 fluorescence to estimate the time-course of axonal loading for both Alexa Fluor 568 and Fluo-4. Because of diffusional re-distribution of the dyes, the fluorescence time-course varied among boutons located at different distances from the soma. After 40 min Alexa Fluor 568 fluorescence stabilized throughout the neuron (up to 600 µm from the cell body). At this time point fluorescence in the soma was 70%–80% of its value at the moment of pipette withdrawal. Therefore, the actual concentrations of the fluorescent dyes in synaptic boutons were ∼25% lower than those in the patch-pipette (i.e., ∼150 µM). In the conditions of our experiments, photobleaching of Alexa Fluor 568 was negligible (less than 1% over 25 frames). (C, D) Estimation of bouton volume using Alexa Fluor 568. Cytosolic bouton volume should be proportional to the total specific Alexa Fluor 568 fluorescence *F_alexa_*. To determine *F_alexa_*, we integrated Alexa Fluor 568 fluorescence in a ROI that completely covers the bouton of interest (C, left panel) and then subtracted the integral background value corresponding to the same ROI. Importantly this estimate of *F_alexa_* does not depend on the exact ROI size and shape, providing that it completely covers the synaptic bouton: (D) rectangular ROIs of increasing size (top) and dependency of the specific integral Alexa Fluor 568 fluorescence on the ROI number, dashed line corresponds to *F_alexa_* estimated in (C). Scale bar 2 µm.
(TIF)

Figure S6 **Comparison of AP-evoked Ca^2+^ fluorescence transients among boutons supplied by single axon.** (A) Superimposed original traces showing variability of Δ*F/F_m_* among boutons recorded in two different axon (five boutons in each experiment). (B) Scaled responses from the same boutons showing similar fluorescence decay rates in boutons from the same axon (average scaled traces are shown in black).
(TIF)

Figure S7
**Non-stationary single compartment model predicts linear relationship between Δ** ***F*** **/** ***F*** **_m_ and** 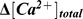 **when 0<Δ** ***F*** **/** ***F*** **_m_<0.6.** Theoretical relationships between AP-evoked peak fluorescence Δ*F/F_m_* and total magnitude of volume averaged presynaptic Ca^2+^ influx 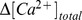 (left panels) and between change of free intracellular Ca^2+^ concentration 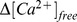 and 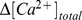 (right panels) in the absence of any endogenous buffers (A) and in the presence of 150 µM Calbindin-D_28K_ (B) or 150 µM Parvalbumin (C). Fluorescence traces at in different conditions were calculated as described in the Text S3. To match the experimental data analysis Δ*F/F_m_* values were obtained by averaging calculated fluorescence response over 10 ms interval immediately after the AP. 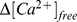 was calculated using 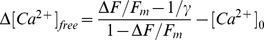 [22] where 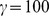 is the dynamic range of Fluo-4 and 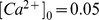 µm is resting Ca^2+^ concentration used in simulations. This modeling predicts that in contrast to 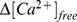, Δ*F/F_m_* (within the experimentally observed range ∼0.1–0.6) should provide a linear readout of the total magnitude of volume averaged AP-evoked presynaptic Ca^2+^ influx 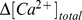.(TIF)

Table S1 **Parameters used in the numerical non-stationary single compartment model.**
(PDF)

Text S1 **Quantification of FM dye (SRC1) de-staining during low-frequency stimulation.**
(PDF)

Text S2 **Measurements of RRP size with high-frequency stimulation.**
(PDF)

Text S3 **Modeling presynaptic Ca^2+^ dynamics using non-stationary single compartment model.**
(PDF)

## Abbreviations

AP: action potential
AZ: active zone
CNS: central nervous system
CV: coefficient of variation
RRP: readily releasable pool
ROI: region of interest
SEM: standard error of the mean
SRC1: SynaptoRedC1
TRP: total recycling pool
VGCC: voltage-gated Ca^2+^ channel
