# Supplementary figures and images for: Independent Regulation of Basal Neurotransmitter Release Efficacy by Variable Ca2+ Influx and Bouton Size at Small Central Synapses
Source: PLoS Biol. 2012 Sep 25;10(9):e1001396. doi: 10.1371/journal.pbio.1001396 (PMC3457933; doi:10.1371/journal.pbio.1001396)

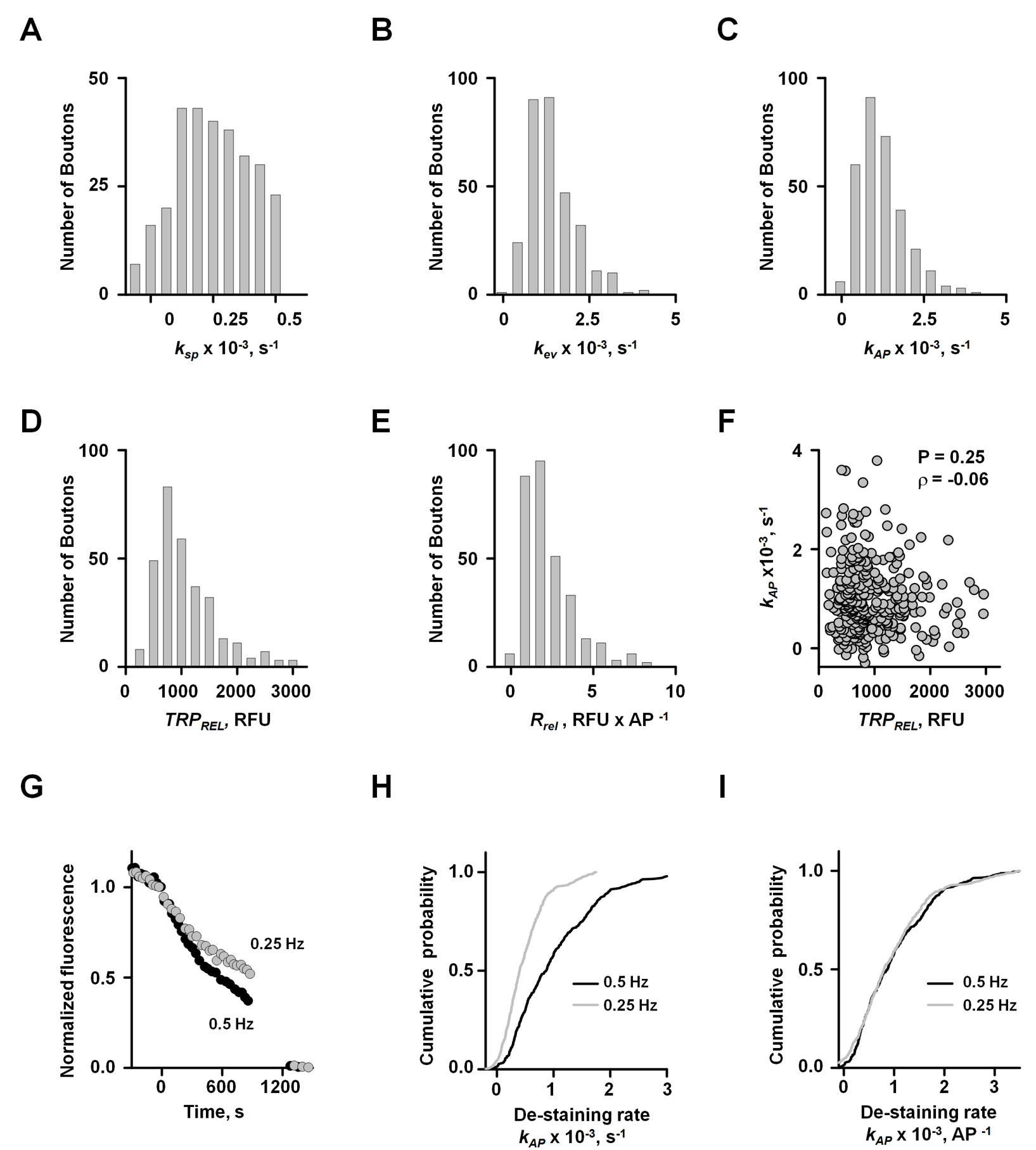

Supplement: Figure S1 — Properties of SRC1 de-staining in individual synaptic boutons during low-frequency stimulation. (A–E) Distribution of SRC1 de-staining parameters recorded in individual boutons (309 boutons from five independent experiments). (A) De-staining rate in the absence of stimulation, ; (B) de-staining rate during 0.5-Hz stimulation, ; (C) Specific AP-evoked de-staining rate ; (D) Relative size of recycling pool of vesicles, calculated as (RFU, relative fluorescence units); (E) Vesicular release rate, ( = 0.5 Hz, stimulation frequency). (F) The AP-evoked de-staining rate does not depend on the TRP size. Correlation coefficient ρ and significance level p (Spearman rank correlation test) are indicated. (G–I) Specific SRC1 de-staining rates calculated per AP are the same during 0.25-Hz and 0.5-Hz stimulation. (G) Average de-staining profiles from two typical experiments at 0.5-Hz and 0.25-Hz stimulation. Distributions of AP-evoked SRC1 de-staining rates at 0.5 Hz (black, 410 boutons from four experiments) and at 0.25 Hz (gray, 544 boutons from four experiments) calculated per second (H) or per AP (I). The absolute AP-evoked SRC1 de-staining rate was lower during 0.25-Hz stimulation (H), whilst the specific SRC1 de-staining rate calculated per AP had the same distribution at 0.25 Hz and 0.5 Hz (I), p = 0.28, Kolmogorov-Smirnov test. (TIF) [file pbio.1001396.s001.tif]

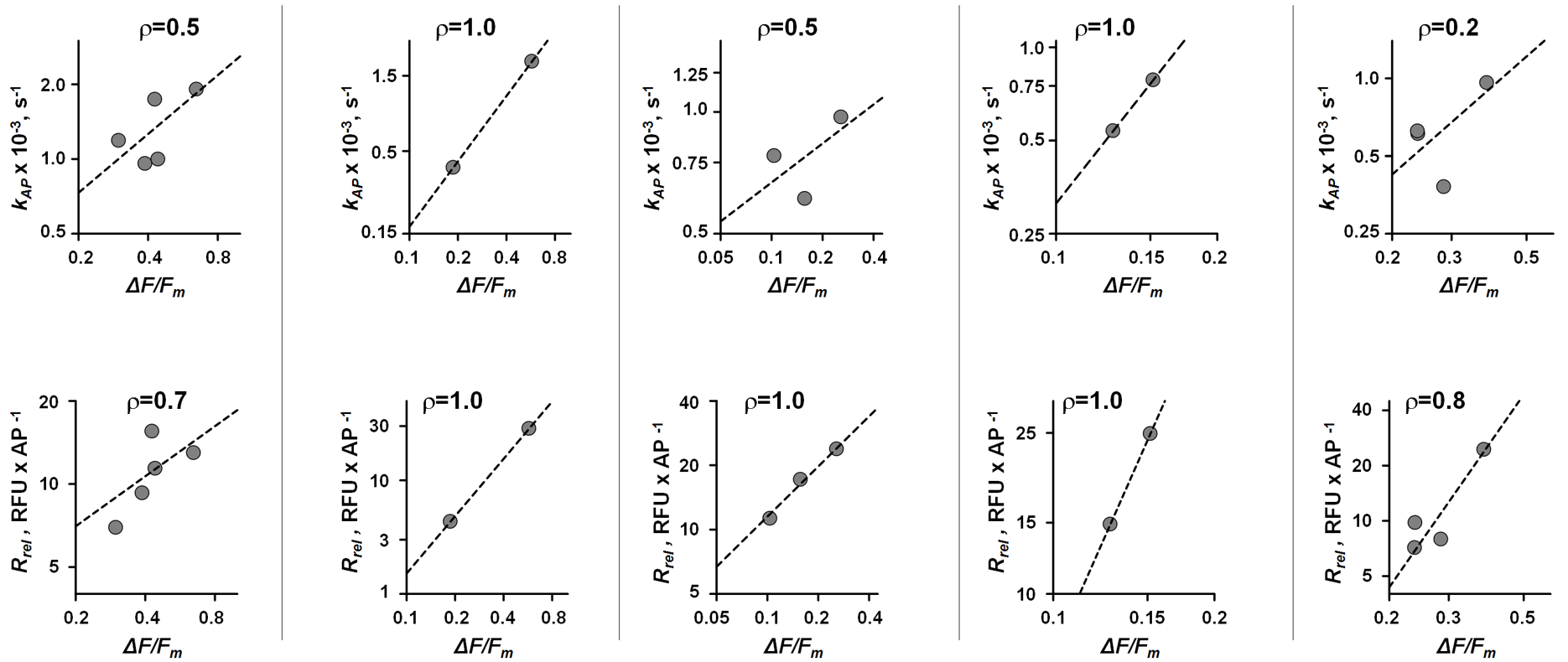

Supplement: Figure S2 — Co-variation of vesicular release rate and AP-evoked presynaptic Ca2+ fluorescence transient: raw data from single axons. Dependencies of AP-evoked SRC1 de-staining rate (top row) and vesicular release rate (bottom row) on the amplitude of AP-evoked presynaptic Ca2+ fluorescence ΔF/Fm in five axons measured in five independent experiments. SRC1 measurements were performed prior to Fluo-4 loading (experimental protocol as in Figure 1, pooled normalized data are shown in Figure 2). Spearman rank correlation coefficients ρ are indicated. Dashed lines show data fits with a power function . (TIF) [file pbio.1001396.s002.tif]

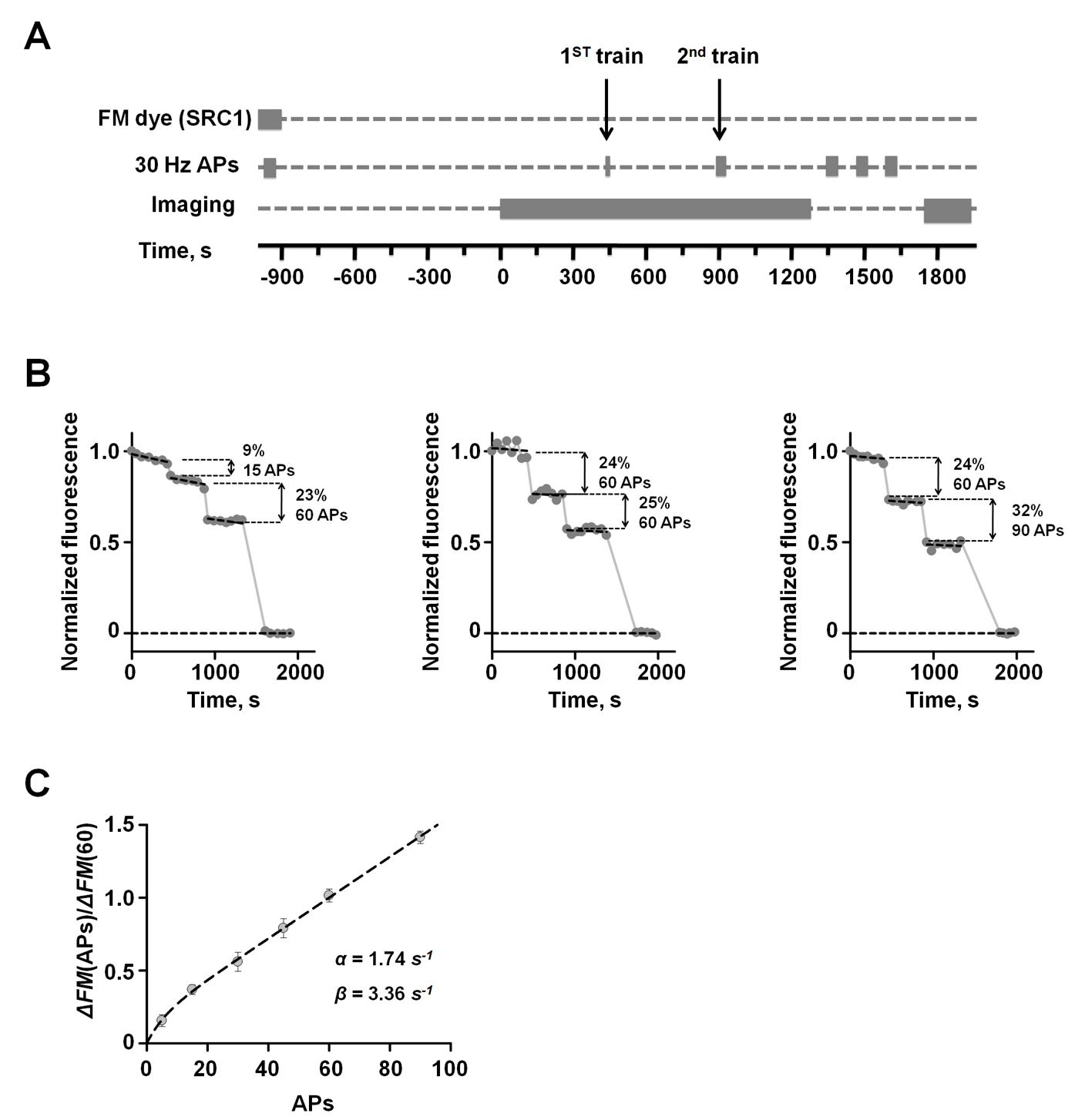

Supplement: Figure S3 — Verification of the protocol for estimating of ratio with short high-frequency stimulation bursts (related to Text S2). (A) Experimental paradigm. After SRC1 loading and washout relative SRC1 fluorescence losses in individual boutons were determined after two trains of 30-Hz stimulation separated by a 7.5-s interval. (B) Example traces recorded in individual boutons using different stimulations protocols. (C) Dependency of the ratio between average SRC1 fluorescence losses after the test train (consisting of n APs) and control train (consisting of 60 APs) on the number of APs in the test train. Dashed line represents a least square data fit using Equation 2.5 from Text S2. Data are mean ± SEM from 150–300 individual boutons from four independent experiments for each condition. (TIF) [file pbio.1001396.s003.tif]

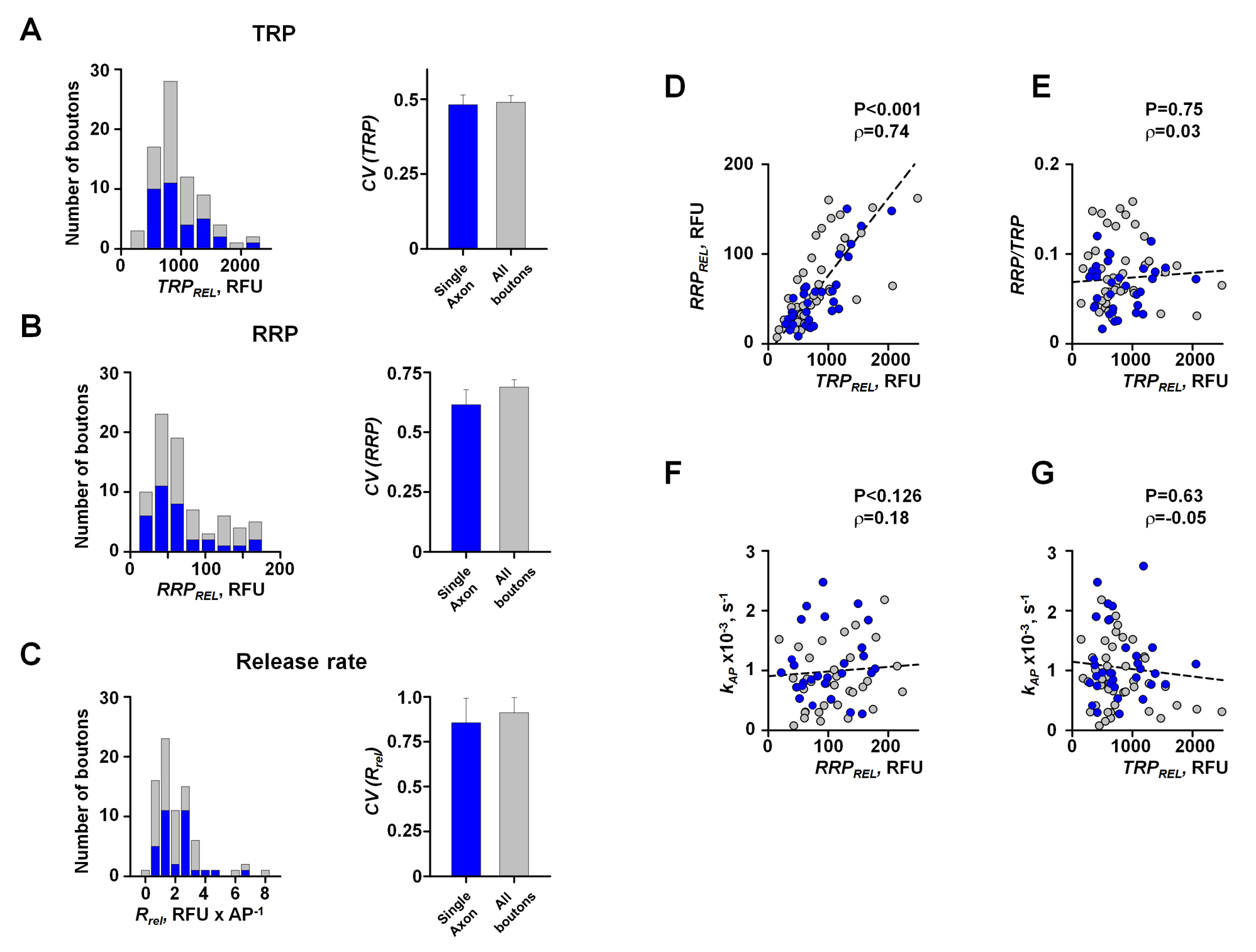

Supplement: Figure S4 — Comparison of functional vesicular pool sizes in synapses supplied by single axons. (A–C) Left panels, frequency histograms of: (A) relative TRP size calculated as total specific loss of SRC1 fluorescence during de-staining experiment: , (B) relative RRP size calculated as the specific fluorescence loss stimulated by a 2-s 30-Hz stimulation divided by the scaling coefficient (see Text S2) , and (C) relative vesicular release rate calculated as from the experiment illustrated in (Figure 3). Blue histogram, boutons supplied by the Alexa loaded axon; gray histogram, all boutons in the field of view. (A–C) Right panels, summary data for variability of: (A) TRP size, (B) RRP size, and (C) . Blue bars, average CVs for synaptic boutons located on single axons; grey bars, average CVs for all boutons recorded in the same experiments. Data are mean ± SEM from 11 independent experiments. (D, E). RRP size scales linearly with the TRP size: relationships between and (D) and between and (E). (F, G) AP-evoked SRC1 de-staining rate does not depend on the RRP size (F) or on the TRP size (G). Data in (D–G) are from the same experiment as illustrated in Figure 3. Blue, data points from boutons supplied by the Alexa loaded axon; grey data points from all boutons in the field of view. Dotted lines in (D–G) show linear regression for all data points. Correlation coefficients ρ and significance levels p (Spearman rank correlation test) are indicated. (TIF) [file pbio.1001396.s004.tif]

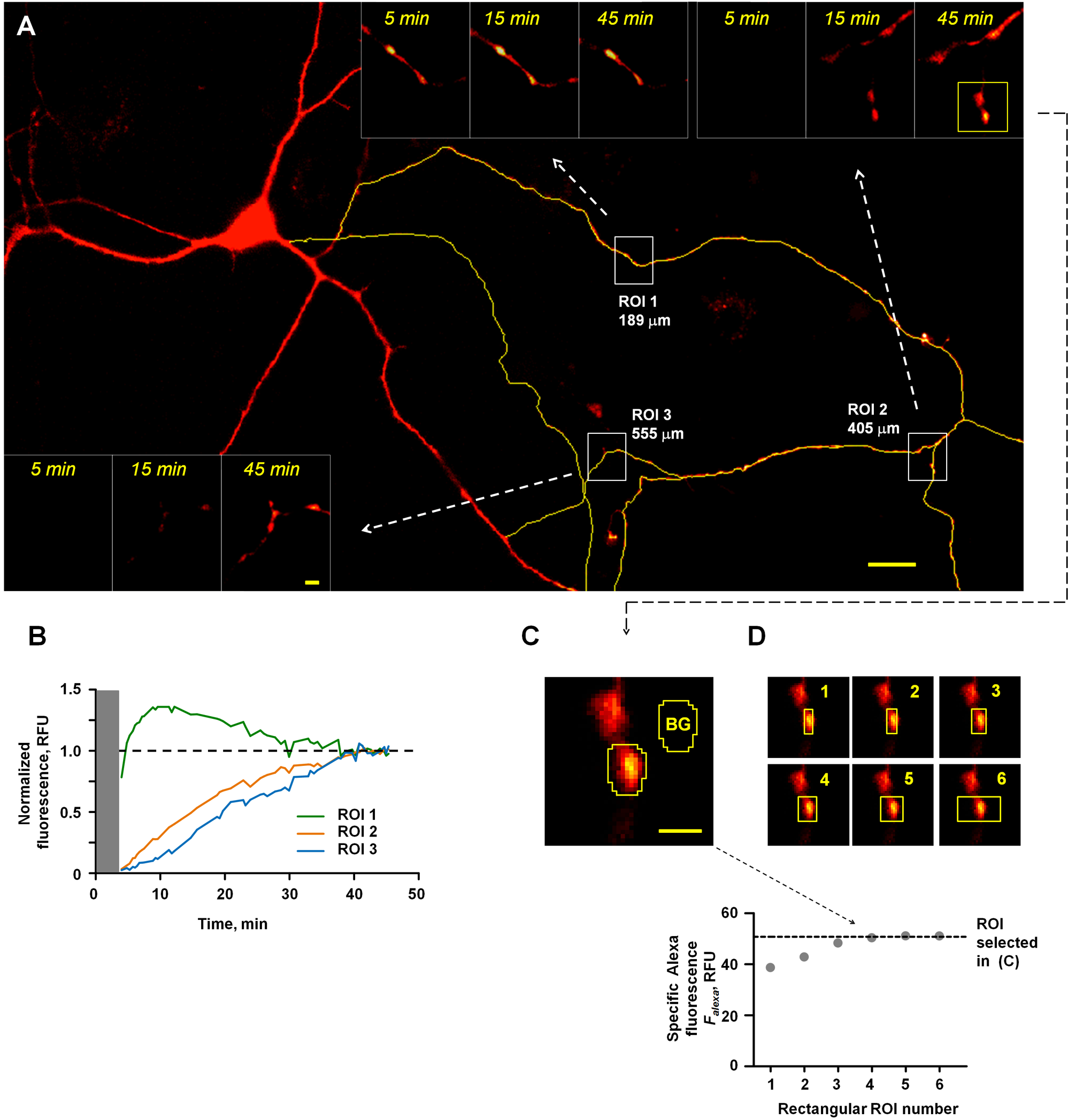

Supplement: Figure S5 — Patch-loading of synaptic boutons with the Ca2+ indicator Fluo-4 and the fluorescent morphological tracer Alexa Fluor 568 and measurements of synaptic bouton size. (A) Fluorescence image of a typical cultured hippocampal neuron 45 min after the beginning of patch-loading with Alexa Fluor 568 and Fluo-4. Note that the patch pipette was withdrawn within 5 min to minimize cytosol dialysis (Methods). The Alexa channel is shown in red, the Fluo-4 channel is not shown, and a reconstructed axon is shown in yellow. Inserts illustrate fluorescence images recorded in axonal boutons located at different distances from the soma (white boxes, ROIs 1, 2, and 3) at 5 min, 15 min, and 45 min after establishing of the whole-cell recording. Scale bars: main figure 20 µm, inserts 2 µm. (B) Time course of Alexa Fluor 568 fluorescence in the selected ROIs from (A). The gray box highlights the interval of the patch-loading (5 min). Since Fluo-4 and Alexa Fluor 568 have indistinguishable intracellular diffusion rates [21] we used Alexa Fluor 568 fluorescence to estimate the time-course of axonal loading for both Alexa Fluor 568 and Fluo-4. Because of diffusional re-distribution of the dyes, the fluorescence time-course varied among boutons located at different distances from the soma. After 40 min Alexa Fluor 568 fluorescence stabilized throughout the neuron (up to 600 µm from the cell body). At this time point fluorescence in the soma was 70%–80% of its value at the moment of pipette withdrawal. Therefore, the actual concentrations of the fluorescent dyes in synaptic boutons were ∼25% lower than those in the patch-pipette (i.e., ∼150 µM). In the conditions of our experiments, photobleaching of Alexa Fluor 568 was negligible (less than 1% over 25 frames). (C, D) Estimation of bouton volume using Alexa Fluor 568. Cytosolic bouton volume should be proportional to the total specific Alexa Fluor 568 fluorescence Falexa. To determine Falexa, we integrated Alexa Fluor 568 fluorescence in a ROI [file pbio.1001396.s005.tif]

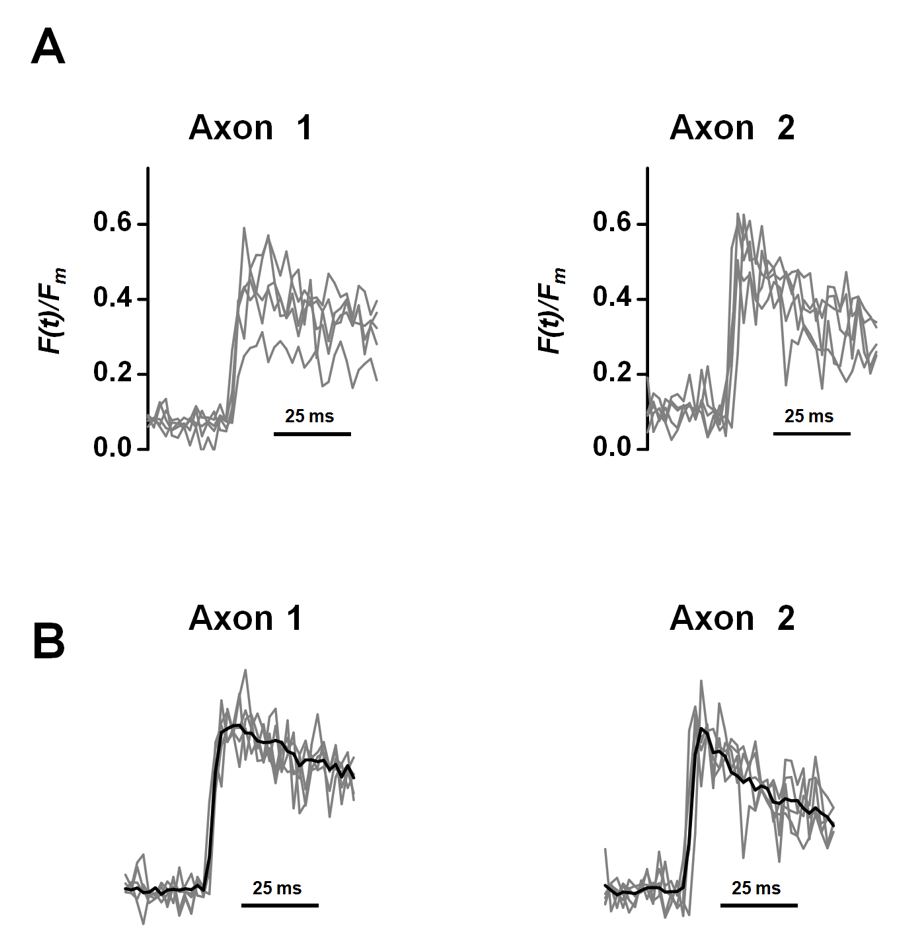

Supplement: Figure S6 — Comparison of AP-evoked Ca2+ fluorescence transients among boutons supplied by single axon. (A) Superimposed original traces showing variability of ΔF/Fm among boutons recorded in two different axon (five boutons in each experiment). (B) Scaled responses from the same boutons showing similar fluorescence decay rates in boutons from the same axon (average scaled traces are shown in black). (TIF) [file pbio.1001396.s006.tif]

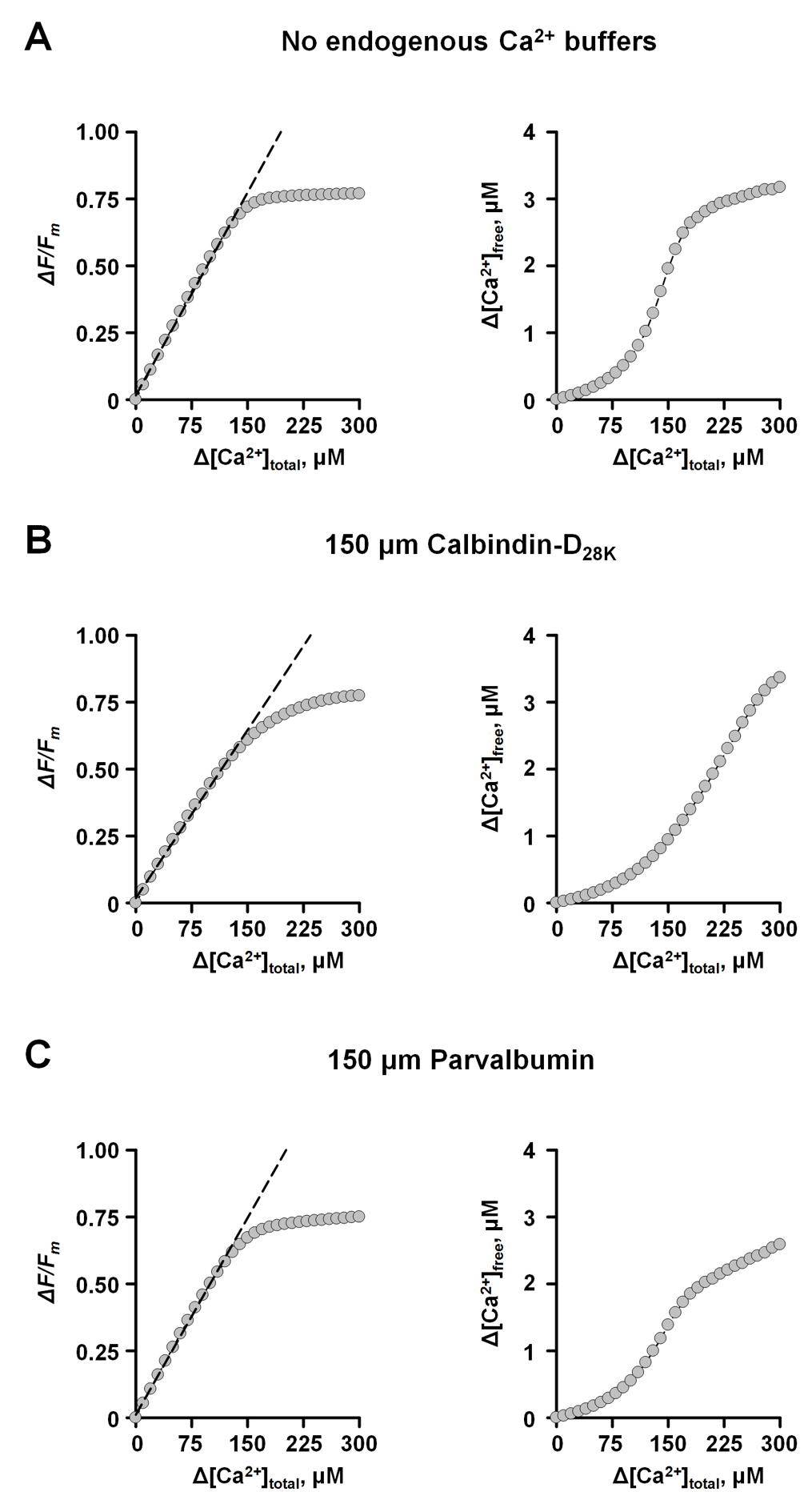

Supplement: Figure S7 — Non-stationary single compartment model predicts linear relationship between Δ F / F m and when 0<Δ F / F m<0.6. Theoretical relationships between AP-evoked peak fluorescence ΔF/Fm and total magnitude of volume averaged presynaptic Ca2+ influx (left panels) and between change of free intracellular Ca2+ concentration and (right panels) in the absence of any endogenous buffers (A) and in the presence of 150 µM Calbindin-D28K (B) or 150 µM Parvalbumin (C). Fluorescence traces at in different conditions were calculated as described in the Text S3. To match the experimental data analysis ΔF/Fm values were obtained by averaging calculated fluorescence response over 10 ms interval immediately after the AP. was calculated using [22] where is the dynamic range of Fluo-4 and µm is resting Ca2+ concentration used in simulations. This modeling predicts that in contrast to , ΔF/Fm (within the experimentally observed range ∼0.1–0.6) should provide a linear readout of the total magnitude of volume averaged AP-evoked presynaptic Ca2+ influx . (TIF) [file pbio.1001396.s007.tif]
