# Supplementary material for: Independent Regulation of Basal Neurotransmitter Release Efficacy by Variable Ca2+ Influx and Bouton Size at Small Central Synapses
Source: PLoS Biol. 2012 Sep 25;10(9):e1001396. doi: 10.1371/journal.pbio.1001396 (PMC3457933; doi:10.1371/journal.pbio.1001396)
Supplement: Table S1 — Parameters used in the numerical non-stationary single compartment model. (PDF) [file pbio.1001396.s008.pdf]

Table S1. Parameters used in the numerical non-stationary single compartment model.

**Model parameter**	**Symbol**	**Value, range tested**
AP duration[1]	σ	0.5 ms
Resting calcium concentration	$[Ca^{2+}]_{rest}$	50 nM*
AP-evoked change of total Ca^{2+} concentration	$\Delta[Ca^{2+}]_{total}$	0 – 300 µM
Ca^{2+} extrusion rate<br>Figure 1 G	k_{rem}	1.0 - 2.0 ms^{-1}
Fluorescent indicator (Fluo-4) Parameters [2,3]		
Ca^{2+} association rate	k_{on}^{I}	0.6 $µM^{-1}$ ms^{-1}
Ca^{2+} dissociation rate	k_{off}^{I}	0.21 ms^{-1}
Total indicator concentration,<br>Figure S2 A and B	$[I]_{total}$	150 - 190 µM
Dynamic range	γ	100
Parvalbumin[4]		
Ca^{2+} association rate	k_{on}^{B}	0.018 $µM^{-1}$ ms^{-1}
Ca^{2+} dissociation rate	k_{off}^{B}	0.00095 ms^{-1}
Dissociation constant	K_{D}^{B}	0.051 µM
Total concentration of Ca^{2+} binding sites	$[B]_{total}$	0 - 300 µM
Calbindin-D_{28K}[5,6]		
Ca^{2+} association rate fast binding sites	$k_{on}^{B_1}$	0.087 $µM^{-1}$ ms^{-1}
Ca^{2+} dissociation rate fast binding sites	$k_{off}^{B_1}$	0.037 ms^{-1}
Dissociation constant fast binding sites	$K_{D}^{B_1}$	0.42 µM
Total concentration of fast Ca^{2+} binding sites	$[B_1]_{total}$	0 - 150 µM
Ca^{2+} association rate slow binding sites	$k_{on}^{B_2}$	0.011 $µM^{-1}$ ms^{-1}
Ca^{2+} dissociation rate slow binding sites	$k_{off}^{B_2}$	0.0026 ms^{-1}
Dissociation constant slow binding sites	$K_{D}^{B_2}$	0.24 µM
Total concentration of slow Ca^{2+} binding sites	$[B_2]_{total}$	0 - 150 µM

*The average resting Ca^{2+} concentration was estimated using the formula

$$[Ca^{2+}]_{rest} = K_D^I \frac{F_0/F_m - 1/\gamma}{1 - F_0/F_m}$$ [3].

Reference List

1. Bischofberger J, Geiger JR, Jonas P (2002) Timing and efficacy of Ca2+ channel activation in hippocampal mossy fiber boutons. J Neurosci 22: 10593-10602.

2. Gee KR, Brown KA, Chen WN, Bishop-Stewart J, Gray D, Johnson I (2000) Chemical and physiological characterization of fluo-4 Ca(2+)-indicator dyes. Cell Calcium 27: 97-106.

3. Maravall M, Mainen ZF, Sabatini BL, Svoboda K (2000) Estimating intracellular calcium concentrations and buffering without wavelength ratioing. Biophys J 78: 2655-2667.

4. Lee SH, Schwaller B, Neher E (2000) Kinetics of Ca2+ binding to parvalbumin in bovine chromaffin cells: implications for [Ca2+] transients of neuronal dendrites. J Physiol 525 Pt 2: 419-432.

5. Nagerl UV, Novo D, Mody I, Vergara JL (2000) Binding kinetics of calbindin-D(28k) determined by flash photolysis of caged Ca(2+). Biophys J 79: 3009-3018.

6. Muller A, Kukley M, Stausberg P, Beck H, Muller W, Dietrich D (2005) Endogenous Ca2+ buffer concentration and Ca2+ microdomains in hippocampal neurons. J Neurosci %19;25: 558-565.
