# Supplementary material for: Independent Regulation of Basal Neurotransmitter Release Efficacy by Variable Ca2+ Influx and Bouton Size at Small Central Synapses
Source: PLoS Biol. 2012 Sep 25;10(9):e1001396. doi: 10.1371/journal.pbio.1001396 (PMC3457933; doi:10.1371/journal.pbio.1001396)
Supplement: Text S1 — Quantification of FM dye (SRC1) de-staining during low-frequency stimulation. (PDF) [file pbio.1001396.s009.pdf]

Text S1. Quantification of FM dye (SRC1) de-staining during low frequency stimulation.

In this section we consider a quantitative model which allows estimating basic presynaptic parameters using SRC1 de-staining profiles recorded in individual synaptic boutons.

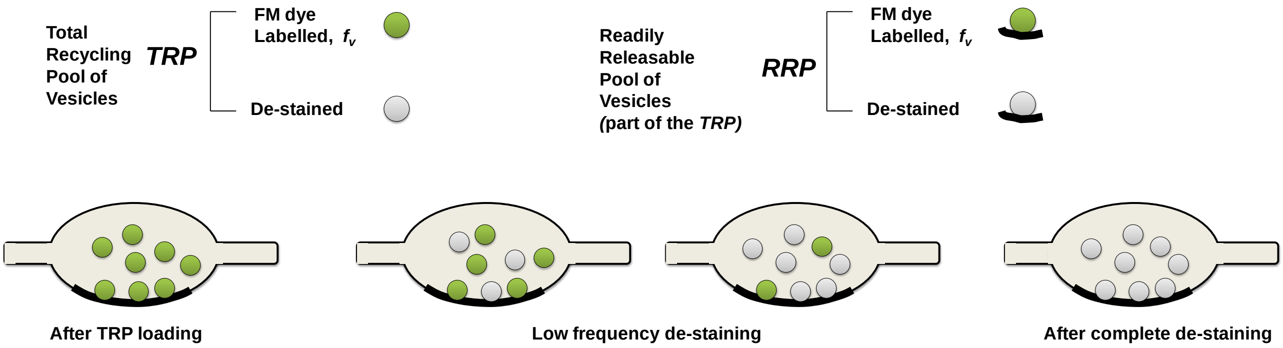

Notation:

f_v specific fluorescence of a single SRC1 labeled vesicle

RRP number of readily-releasable vesicles

TRP total number of recycling vesicles

p_v average release probability of individual vesicles within RRP

$F_{FM}(t)$ specific vesicular FM dye fluorescence intensity in individual synaptic boutons

Model assumptions:

In general, FM dye de-staining profiles have complex shapes that depend on the type of FM dye used and on loading/stimulation protocols [1–5]. It has however been shown that vesicles within RRP and TRP co-exist in a dynamic equilibrium [6,7]. Therefore we hypothesized that, during low frequency (0.5 Hz) stimulation, there is enough time for

newly endocytosed fluorescence-free vesicles to re-equilibrate between the TRP and RRP between successive APs. To test this hypothesis we compared SRC1 de-staining at 0.5 Hz and 0.25 Hz. If SRC1 de-staining was significantly affected by depletion of RRP vesicles or by preferential recycling of RPP vesicles (for example as has been reported after bursts of high frequency stimulation [7]), then the SRC1 de-staining per AP should be higher at 0.25 Hz than at 0.5 Hz. However, no such difference between SRC1 de-staining rates at these two frequencies was found when k_{AP} was normalized by the number of APs (Figure S1 G - I). We therefore conclude that, during low frequency stimulation, fluorescence-labeled and fluorescence-free vesicles were equally distributed between RRP and TRP.

We then obtain:

$$\frac{dF_{FM}(t)}{dt} = -\nu \cdot N_{AP} \cdot f_v \cdot \frac{F_{FM}(t)}{\Delta F_{FMtotal}} \tag{1.1}$$

where $\Delta F_{FMtotal} = TRP \cdot f_v$ is the specific fluorescence of FM dye labeled vesicles corresponding to completely labeled TRP, and N_{AP} is the average number of vesicles released during a single AP. Under the binomial model [8–10], the average number of vesicles released during a single AP (i.e. vesicular release rate) follows the relationship: $N_{AP} = RRP \cdot p_v$. This yields:

$$\frac{dF_{FM}(t)}{dt} = -\nu \cdot [RRP/TRP] \cdot p_v \cdot F_{FM}(t)$$

and

$$F_{FM}(t) = \Delta F_{FMtotal} \cdot \exp(-\nu \cdot [RRP/TRP] \cdot p_v \cdot t)$$

Thus the experimentally determined AP-specific SRC1 de-staining rate can be expressed as:

$$k_{AP} = \nu \cdot \left[RRP/TRP\right] p_v \tag{1.2}$$

Consequently, another experimentally determined parameter – the vesicular release rate ($R_{rel} = \frac{\Delta F_{FMtotal} k_{AP}}{v}$) – is proportional to the average number of vesicles released during a single AP - $R_{rel} \propto N_{AP}$:

$$R_{rel} = \frac{\Delta F_{FMtotal} k_{AP}}{v} = TRP \cdot f_v \cdot [RRP/TRP] \cdot p_v = f_v \cdot RRP \cdot p_v = f_v \cdot N_{AP}$$

Reference List

1. Klingauf J, Kavalali ET, Tsien RW (1998) Kinetics and regulation of fast endocytosis at hippocampal synapses. Nature 394: 581-585.

2. Vanden BP, Klingauf J (2006) Synaptic vesicles in rat hippocampal boutons recycle to different pools in a use-dependent fashion. J Physiol 572: 707-720.

3. Mozhayeva MG, Sara Y, Liu X, Kavalali ET (2002) Development of vesicle pools during maturation of hippocampal synapses. J Neurosci 22: 654-665.

4. Murthy VN, Sejnowski TJ, Stevens CF (1997) Heterogeneous release properties of visualized individual hippocampal synapses. Neuron 18: 599-612.

5. Waters J, Smith SJ (2002) Vesicle pool partitioning influences presynaptic diversity and weighting in rat hippocampal synapses. J Physiol 541: 811-823.

6. Murthy VN, Stevens CF (1999) Reversal of synaptic vesicle docking at central synapses. Nat Neurosci 2: 503-507.

7. Pyle JL, Kavalali ET, Piedras-Renteria ES, Tsien RW (2000) Rapid reuse of readily releasable pool vesicles at hippocampal synapses. Neuron 28: 221-231.

8. Schneggenburger R, Sakaba T, Neher E (2002) Vesicle pools and short-term synaptic depression: lessons from a large synapse. Trends Neurosci 25: 206-212.

9. Christie JM, Jahr CE (2006) Multivesicular release at Schaffer collateral-CA1 hippocampal synapses. J Neurosci 26: 210-216.

10. Oertner TG, Sabatini BL, Nimchinsky EA, Svoboda K (2002) Facilitation at single synapses probed with optical quantal analysis. Nat Neurosci 5: 657-664.
