# Supplementary material for: Independent Regulation of Basal Neurotransmitter Release Efficacy by Variable Ca2+ Influx and Bouton Size at Small Central Synapses
Source: PLoS Biol. 2012 Sep 25;10(9):e1001396. doi: 10.1371/journal.pbio.1001396 (PMC3457933; doi:10.1371/journal.pbio.1001396)
Supplement: Text S2 — Measurements of RRP size with high-frequency stimulation. (PDF) [file pbio.1001396.s010.pdf]

Text S2. Measurements of RRP size with high frequency stimulation.

In this section we consider and experimentally verify a quantitative model which allows estimating the relative RRP size in individual syanptic boutons using SRC1 de-staining kinetics during high frequency AP stimulation.

The size of the RRP of vesicles in CNS synapses can be estimated either by brief application of hypertonic solutions (e.g. sucrose) or by high frequency AP stimulation [1,2]. Although both methods deplete the same pool of vesicles, the estimates of the RRP size can vary depending on the protocol used [3–6]. An important source of discrepancy is active vesicle replenishment during RRP depletion, which can, to a first approximation, be dealt with by assuming that refilling of the RRP follows first-order kinetics [2,7–10]. Here we further explored the model of RRP dynamics originally described in Ref. [9] and verified in Ref. [3]. The model assumes a RRP of a fixed size containing individual release sites that fill independently with first order kinetics. Then, the probability p_{oc} that any of these release sites is occupied by a vesicle during the train of APs follows the equation:

$$\frac{dp_{oc}(t)}{dt} = \alpha \cdot (1 - p_{oc}(t)) - \beta \cdot p_{oc}(t) \qquad (2.1)$$

where α (s^{-1}) is the rate of RRP refilling and β (s^{-1}) is the average rate of vesicular release during AP train (β includes both synchronous and asynchronous AP-evoked release).

Because newly endocytosed vesicles do not significantly contribute to vesicular release during short bursts of high frequency stimulation [11,12] the SRC1 de-staining kinetics should follow the equation:

$$\frac{dF_{FM}(t)}{dt} = -RRP \cdot f_v \cdot \beta \cdot p_{oc}(t)$$

where f_v is the specific fluorescence of a single SRC1 labeled vesicle. By taking into account that the specific fluorescence of FM dye labeled vesicles (corresponding to completely labeled TRP) is $\Delta F_{FMtotal} = TRP \cdot f_v$ we obtain:

$$\frac{dF_{FM}(t)}{dt} = -\Delta F_{FMtotal} \cdot \frac{RRP}{TRP} \cdot \beta \cdot p_{oc}(t) \tag{2.2}$$

Without making any further assumptions, by simultaneously integrating (2.1) and (2.2) and by taking into account that $p_{oc}(0) = 1$ and $F_{FM}(0) = \Delta F_{FMtotal}$ we obtain that cumulative SRC1 fluorescence loss during a short high frequency train of APs $\Delta FM(t) \equiv \Delta F_{FMtotal} - F_{FM}(t)$ should follow the equation:

$$\Delta FM(t) = [RRP / TRP] \cdot \Delta F_{FMtotal} \cdot \varphi(\alpha, \beta, t) \tag{2.3}$$

where

$$\varphi(\alpha, \beta, t) = \frac{\alpha\beta}{\alpha + \beta} t + \frac{\beta^2}{(\alpha + \beta)^2} (1 - \exp(-(\alpha + \beta)t)) \tag{2.4}$$

Thus the model predicts that SRC1 fluorescence loss triggered by a burst of high frequency stimulation should be proportional to the true RRP / TRP ratio in each recorded bouton. To determine the coefficient of proportionality $\varphi(\alpha, \beta, t)$ we estimated the average values for parameters α and β across the synaptic population under study. To achieve this we concurrently measured the relative drop of SRC1 fluorescence in individual synaptic boutons in response to a control burst of 60 APs at 30 Hz ($\Delta FM(60 APs)$) and to a test burst consisting of a variable number of APs at 30 Hz ($\Delta FM(N\ APs)$) (Figure S3). The interval between the two bursts was set at 5.0 - 7.5 min, which has previously been shown to be sufficient for complete equilibration of FM dye labeled vesicles between RRP and TRP [4]. We confirmed that delivering the control burst twice with this interval produced identical fluorescence drops (24.0 ± 0.6 % at the

first application and 24.4 ± 0.6 % at the second application, n = 246 boutons). We then alternated the order of the test and control bursts in different experiments and plotted the ratio $\Delta FM(N\ \ APs)/\Delta FM(60APs)$ against the number of APs in the test burst (Figure S3 C). In agreement with previous reports [3,13] we observed no significant depression in the rate of SRC1 fluorescence loss even after 3 seconds of 30 Hz stimulation, arguing that the rate of RRP refilling during 30 Hz stimulation is comparable to the rate of vesicular release. The ratio $\Delta FM(N\ \ APs)/\Delta FM(60APs)$ does not depend on the absolute vesicular pool size:

$$\frac{\Delta FM(N\ \ APs)}{\Delta FM(60APs)} = \frac{\alpha(\alpha+\beta)t_{N\ \ APs} + \beta(1-\exp(-(\alpha+\beta)t_{N\ \ APs})}{\alpha(\alpha+\beta)t_{60APs} + \beta(1-\exp(-(\alpha+\beta)t_{60APs}))} \qquad (2.5).$$

Therefore fitting the data (Figure S3 C) using Equation 2.5 allowed us to estimate the average RRP refilling rate (α = 1.74 s^{-1}) and the average vesicular release rate (β = 3.36 s^{-1}).

By substituting these values into Equation 2.3, and by taking into account that 2 sec 30 Hz stimulation on average lead to ~ 24% relative fluorescence loss, we obtained an estimate of the correction factor $\varphi(\alpha,\beta,t_{60APs})$ for the 60 AP 30 Hz train (~ 2.74) and then calculated the true average ratio RRP/TRP ~ 0.088. Importantly, this estimate agrees very well with a recent report where the RRP/TRP ratio was estimated in the same preparation by using an alternative method based on rapid imaging of pH sensitive exocytosis indicator vGlut-1-pHluorin: RRP/TRP ~ 0.06 – 0.07) [13].

By setting $\varphi(\alpha,\beta,t_{N\ \ AP})$ = 1 we also estimated that, in our experimental conditions, the RRP should be depleted by approximately 15 APs at 30 Hz. Importantly, however, the relative SRC1 fluorescence loss at each bouton is proportional to the RRP/TRP ratio (Equation 2.3), and therefore one can use any arbitrary number of APs in a 30 Hz train to compare relative RRP sizes among individual synapses. In order to increase the signal

to noise ratio (especially for boutons with low RRP/TRP values) we decided to use a 60 AP train, and then estimate the true RRP/TRP value as: $\Delta FM(60APs)$ / 2.74.

Comparison of RRP/TRP ratios measured in individual boutons may be affected by errors arising from possible inter-synaptic variations in the RRP refilling rate α and the vesicular release rate β. However, measurements of the relative RRP size with a 30 Hz train of 60 APs should be relatively insensitive to such variation of parameters α and β. Indeed, using Equation 2.3, we estimate that a two-fold variation of either of these two parameters will lead only to 25 - 35 % change of the apparent RRP/TRP ratio.

Reference List

1. Rosenmund C, Stevens CF (1996) Definition of the readily releasable pool of vesicles at hippocampal synapses. Neuron 16: 1197-1207.

2. Schneggenburger R, Meyer AC, Neher E (1999) Released fraction and total size of a pool of immediately available transmitter quanta at a calyx synapse. Neuron 23: 399-409.

3. Stevens CF, Williams JH (2007) Discharge of the readily releasable pool with action potentials at hippocampal synapses. J Neurophysiol 98: 3221-3229.

4. Pyle JL, Kavalali ET, Piedras-Renteria ES, Tsien RW (2000) Rapid reuse of readily releasable pool vesicles at hippocampal synapses. Neuron 28: 221-231.

5. Schikorski T, Stevens CF (2001) Morphological correlates of functionally defined synaptic vesicle populations. Nat Neurosci 4: 391-395.

6. Moulder KL, Mennerick S (2005) Reluctant vesicles contribute to the total readily releasable pool in glutamatergic hippocampal neurons. J Neurosci 25: 3842-3850.

7. Stevens CF, Wesseling JF (1998) Activity-dependent modulation of the rate at which synaptic vesicles become available to undergo exocytosis. Neuron 21: 415-424.

8. Stevens CF, Sullivan JM (1998) Regulation of the readily releasable vesicle pool by protein kinase C. Neuron 21: 885-893.

9. Wesseling JF, Lo DC (2002) Limit on the role of activity in controlling the release-ready supply of synaptic vesicles. J Neurosci 22: 9708-9720.

10. Stevens CF, Tsujimoto T (1995) Estimates for the pool size of releasable quanta at a single central synapse and for the time required to refill the pool. Proc Natl Acad Sci U S A 92: 846-849.

11. Harata NC, Choi S, Pyle JL, Aravanis AM, Tsien RW (2006) Frequency-dependent kinetics and prevalence of kiss-and-run and reuse at hippocampal synapses studied with novel quenching methods. Neuron 49: 243-256.

12. Balaji J, Ryan TA (2007) Single-vesicle imaging reveals that synaptic vesicle exocytosis and endocytosis are coupled by a single stochastic mode. Proc Natl Acad Sci U S A 104: 20576-20581.

13. Ariel P, Ryan TA (2010) Optical mapping of release properties in synapses. Front Neural Circuits 4.
