# Supplementary material for: Independent Regulation of Basal Neurotransmitter Release Efficacy by Variable Ca2+ Influx and Bouton Size at Small Central Synapses
Source: PLoS Biol. 2012 Sep 25;10(9):e1001396. doi: 10.1371/journal.pbio.1001396 (PMC3457933; doi:10.1371/journal.pbio.1001396)
Supplement: Text S3 — Modeling presynaptic Ca2+ dynamics using non-stationary single compartment model. (PDF) [file pbio.1001396.s011.pdf]

Text S3. Modeling presynaptic Ca^{2+} dynamics using non-stationary single compartment model

The non-stationary model of presynaptic Ca^{2+} dynamics has been previously described by [1,2]. Briefly, the model assumes that presynaptic Ca^{2+} dynamics depend on the Ca^{2+} entry rate j_{Ca}, binding-unbinding reactions with the endogenous buffers B_i and Ca^{2+} indicator I, and Ca^{2+} removal. The latter is a complex process involving diffusion, pumping out and/or sequestration; generally, it could be approximated by a first-order reaction [3,4] with rate $P = k_{rem}\left(\left[Ca^{2+}\right]-\left[Ca^{2+}\right]_{rest}\right)$. These considerations are reflected in the system of equations below, where the brackets denote concentrations, and the superscript indices of the reaction rate constants denote endogenous Ca^{2+} buffers B_i or the indicator I.

$$\frac{d\left[Ca^{2+}\right]}{dt} = j_{Ca} + k_{off}^{I}[CaI] - k_{on}^{I}[Ca^{2+}][I] + \sum_i \left(k_{off}^{B_i}[CaB_i] - k_{on}^{B_i}[Ca^{2+}][B_i]\right) - k_{rem}\left(\left[Ca^{2+}\right]-\left[Ca^{2+}\right]_{rest}\right)$$

$$\frac{d[I]}{dt} = k_{off}^{I}[CaI] - k_{on}^{I}[Ca^{2+}][I]$$

$$\frac{d[B_i]}{dt} = k_{off}^{B_i}[CaB_i] - k_{on}^{B_i}[Ca^{2+}][B_i]$$

The AP-dependent Ca^{2+} influx time course j_{Ca} was approximated by the Gaussian function $j_{Ca} = \frac{\Delta[Ca^{2+}]_{total}}{\sigma\sqrt{2\pi}}\exp\left(-\frac{(t-t_{AP})^2}{2\sigma^2}\right)$ where t_{AP} denotes the time of the AP and $\Delta[Ca^{2+}]_{total}$ denotes the total time integral of the volume averaged presynaptic Ca^{2+} entry. The mass conservation rules for this system are: $[I]_{total} = [I]+[CaI]$, $[B_i]_{total} = [B_i]+[CaB_i]$.

For each set of simulations we numerically solved the above model using the adaptive step-size Runge-Kutta algorithm and calculated the Fluo-4 fluorescence profile normalized to maximal fluorescence of the saturated indicator:

$\frac{F(t)}{F_m} = \frac{[CaI](t) \cdot \gamma + [I](t)}{[I]_{total} \cdot \gamma}$ (where γ ~ 100 is the dynamic range of Fluo-4). The parameters used in the numerical model are summarized in Table S1.

Reference List

1. Scott R, Rusakov DA (2006) Main determinants of presynaptic Ca2+ dynamics at individual mossy fiber-CA3 pyramidal cell synapses. J Neurosci 26: 7071-7081.

2. Sabatini BL, Regehr WG (1998) Optical measurement of presynaptic calcium currents. Biophys J 74: 1549-1563.

3. Jackson MB, Redman SJ (2003) Calcium dynamics, buffering, and buffer saturation in the boutons of dentate granule-cell axons in the hilus. J Neurosci 23: 1612-1621.

4. Matveev V, Zucker RS, Sherman A (2004) Facilitation through buffer saturation: constraints on endogenous buffering properties. Biophys J 86: 2691-2709.
